# The Impact of [C16Pyr][Amp] on the Aggressiveness in Breast and Prostate Cancer Cell Lines

**DOI:** 10.3390/ijms21249584

**Published:** 2020-12-16

**Authors:** Filipa Quintela Vieira, Ângela Marques-Magalhães, Vera Miranda-Gonçalves, Ricardo Ferraz, Mónica Vieira, Cristina Prudêncio, Carmen Jerónimo, Regina Augusta Silva

**Affiliations:** 1 Research Centre in Health and Environment (CISA), School of Health (ESS), Polytechnic Institute of Porto (P.PORTO), 4200-072 Porto, Portugal; afv@eu.ipp.pt (F.Q.V.); magalhaes.angela94@gmail.com (Â.M.-M.); rferraz@ess.ipp.pt (R.F.); mav@ess.ipp.pt (M.V.); cprudencio@ess.ipp.pt (C.P.); 2 Department of Pathological, Cytological and Thanatological Anatomy, ESS|P.PORTO, 4200-072 Porto, Portugal; 3 Cancer Biology and Epigenetics Group, IPO Porto Research Center (CI-IPOP), Portuguese Oncology Institute of Porto (IPO Porto), 4200-072 Porto, Portugal; Vera.Miranda.Goncalves@ipoporto.min-saude.pt; 4 LAQV-REQUIMTE, Departamento de Química e Bioquímica, Faculdade de Ciências, Universidade do Porto, 4169-007 Porto, Portugal; 5 Ciências Químicas e das Biomoléculas, ESS|P.PORTO, 4200-072 Porto, Portugal; 6 Instituto de Investigação e Inovação em Saúde (i3S), Universidade do Porto, 4200-072 Porto, Portugal; 7 Department of Pathology and Molecular Immunology, Institute of Biomedical Sciences Abel Salazar (ICBAS), University of Porto, 4050-313 Porto, Portugal

**Keywords:** breast cancer, prostate cancer, ionic liquids, quinoxalines, treatment

## Abstract

Breast (BrCa) and prostate (PCa) cancers are the most common malignancies in women and men, respectively. The available therapeutic options for these tumors are still not curative and have severe side effects. Therefore, there is an urgent need for more effective antineoplastic agents. Herein, BrCa, PCa, and benign cell lines were treated with two ionic liquids and two quinoxalines and functional experiments were performed—namely cell viability, apoptosis, cytotoxicity, and colony formation assays. At the molecular level, an array of gene expressions encompassing several molecular pathways were used to explore the impact of treatment on gene expression. Although both quinoxalines and the ionic liquid [C2OHMIM][Amp] did not show any effect on the BrCa and PCa cell lines, [C16Pyr][Amp] significantly decreased cell viability and colony formation ability, while it increased the apoptosis levels of all cell lines. Importantly, [C16Pyr][Amp] was found to be more selective for cancer cells and less toxic than cisplatin. At the molecular level, this ionic liquid was also associated with reduced expression levels of *CPT2*, *LDHA*, *MCM2,* and *SKP2*, in both BrCa and PCa cell lines. Hence, [C16Pyr][Amp] was shown to be a promising anticancer therapeutic agent for BrCa and PCa cell lines.

## 1. Introduction

Cancer is one of the leading causes of disease-related death worldwide. In particular, breast (BrCa) and prostate (PCa) cancer are the first and second most common cancers in women and men, respectively [1]. Concerning BrCa, more than 70% are positive for estrogen receptor expression and, from these, approximately 65% are progesterone receptor-positive and candidates for endocrine therapies [2,3]. Although these tumors are less aggressive and present a better outcome compared to hormone-negative BrCa [4], 30–50% of patients present disease relapse [5]. Moreover, metastatic BrCa remains an incurable disease with a median overall survival of approximately two to three years and a five-year survival rate of only 25% of BrCa patients [6]. Indeed, BrCa remains the foremost cause of cancer-related death in women due to the development of recurrent and/or metastatic disease [1,7]. Likewise, 30% of PCa patients with advanced disease that initially respond to androgen deprivation therapy develop a lethal castration-resistant PCa after 18–24 months [8]. Although these patients might be treated with secondary hormonal therapeutic agents, they inevitably develop therapy resistance and relapse [9]. In the worst-case scenario of metastatic castration-resistant PCa, even after standard therapy with chemotherapy, the disease progresses after few months, with an overall survival (OS) lower than two years [10,11].

Ionic liquids (ILs) and quinoxalines have emerged has potential anticancer drugs [12,13]. ILs are organic salts with a melting point below 100 °C which have been receiving increasing interest, not only in the scientific community but also in industry [14]. ILs are exclusively made up of ions, giving rise to many possible cation–anion combinations, and have high chemical stability [15,16]. Moreover, these multiple combinations allow the adjustment of interactions for a variety of tunable applications. For example, in the case of the polarity and hydrophobicity of ILs, these properties could be adjusted to reach the required effect, making them attractive for drug development and therapeutic treatment [17]. This has led to a wide range of applications in chemistry, biotechnology, pharmaceutics, and medicine [18,19,20]. In the case of ionics based on ampicillin, they have been described to display antiproliferative activity against human cancer cell lines with very low cytotoxicity to normal human cells [12]. Herein, we focused on members from the imidazolium and pyridinium classes as anticancer compounds: [C2OHMIM][Amp] (3-(2-hydroxyethyl)-1-methyl-1H-imidazol-3-ium ampicillinate) and [C16Pyr][Amp] (1-hexadecylpyridin-1-ium ampicillinate) (Figure S1).

Quinoxalines are chemical compounds whose structures are similar to quinolone antibiotics. Quinoxaline derivates are synthetic heterocyclic compounds formed by the fusion of two aromatic rings, i.e., benzene and pyrazine, with a nitrogen atom replacing carbons in the naphthalene ring [21]. Although rare in a natural state, they have an easy synthesis and present several biological properties—namely anti-inflammatory, antibacterial, antifungal, antiviral, antioxidant, and anticancer activities [13,21]. In fact, quinoxaline-1,4-dioxide and 2-methylquinoxaline-1,4-dioxide (Figure S1) were previously described as promising antibacterial agents [13] and also revealed anticancer properties with low human cytotoxicity [22].

Considering the promising applications of both ILs and quinoxalines in biomedical research and the pharmaceutical industry, we aimed to investigate the effect of these synthetic compounds—in particular, two ILs, one with imidazolium ([C2OHMIM][Amp]) and the other with pyridinium ([C16Pyr][Amp]) cations, as well as quinoxaline-1,4-dioxide and 2-methylquinoxaline-1,4-dioxide—in BrCa and PCa cell lines with a malignant phenotype. These four compounds were chosen as they have shown promising results in previous publications [12,22].

## 2. Results

### 2.1. [C16Pyr][Amp] Synthetic Compound Displayed Low Half-Maximal Inhibitory Concentration (IC_50_) Values

Ten immortalized epithelial cell lines from the breast and prostate were used to assess the impact of four compounds—namely two quinoxalines (i.e., quinoxaline-1,4-dioxide and 2-methylquinoxaline-1,4-dioxide) and two ILs based on ampicillin (i.e., [C16Pyr][Amp] and [C2OHMIM][Amp]). The BrCa cell lines used were HTB22 (MCF-7), HTB133 (T-47D), HCC1937, and MDA-MB-231, while 22Rv1, LNCaP, Du145, and PC-3 were the PCa cell lines chosen. The remaining two cell lines were benign and used as controls: MCF-10A (breast) and RWPE-1 (prostate).

For each compound, the half-maximal inhibitory concentration (IC_50_) of each cell line and the dose–response curves were evaluated with 3-(4,5-dimethylthiazol-2-yl)-2,5-diphenyltetrazolium-bromide (MTT) assay. The IC_50_ of the compounds was determined by exposing each cell line to different concentrations of the compounds (Figure S2). [C16Pyr][Amp] exhibited the most promising results, reducing the viability of all cell lines (Table 1 and Figure S2). Nonetheless, neither of the quinoxalines or [C2OHMIM][Amp] showed a significant effect on the viability of the BrCa (Figure S2A) or PCa (Figure S2B) cell lines with the range of concentrations used. Importantly, the IC_50_ value of [C16Pyr][Amp] for the BrCa hormone-independent and PCa castration-resistant cell lines HCC1927, MDA-MB-231, and PC-3, which are representative of the most aggressive tumor phenotypes, was lower than the IC_50_ value for the corresponding benign cell line (MCF-10A and RWPE). Considering these results, phenotypic assays were only performed with the IL [C16Pyr][Amp], at both the IC_50_ and at the double IC_50_ concentrations for each cell line.

### 2.2. [C16Pyr][Amp] Exhibited Higher Selectivity toward Tumor Cells Compared to Cisplatin

Cisplatin was the first FDA-approved platinum compound for cancer treatment in 1978, being a well-known chemotherapeutic agent widely used for several tumors in the clinic [23,24]. Cisplatin is a DNA-intercalating agent that cross-links and denatures DNA strands, leading to cytotoxic effects [25]. Moreover, it produces DNA adducts and induces oxidative stress and DNA damage that interfere with RNA transcription and DNA synthesis, triggering cell cycle arrest and apoptosis [25,26,27]. In BrCa, cisplatin has been used in triple-negative BrCa patients [28] and has also been tested in clinical trials involving other tumor types [29,30]. In PCa, several clinical trials using cisplatin alone or in combination have shown its antitumor activity, with partial and complete responses and improved progression-free and overall survival [31,32,33,34,35,36,37].

Knowing the relevance of this chemotherapeutic agent in the clinic, we used this drug as a control. In our study, [C16Pyr][Amp] presented lower IC_50_ values than cisplatin (*p* = 0.0039) in both BrCa and PCa cell lines (Table 1 and Figure S3). Moreover, as previously described, cisplatin displayed a lower IC_50_ value in benign cell lines (MCF-10A and RWPE) than in tumor cells, indicating that cisplatin is non-specific for tumor cells and induces toxicity to normal cells [38,39,40]. Indeed, the cisplatin selectivity index (SI) was rather limited in most of the tumor cell lines (SI < 1), with the exception of MDA-MB-231 (SI = 4.37) and DU145 (SI = 8.20) (Table 2). Hence, [C16Pyr][Amp] displayed comparatively higher SI values in all cell lines in both tumor models than cisplatin, although without statistical significance (*p* = 0.1094). Regarding the BrCa cell lines, [C16Pyr][Amp] presented the highest SI value for MDA-MB-231 (SI = 11.00). This value is 2.5-fold the SI value found for cisplatin, which is a promising result since MDA-MB-231 is an aggressive cell line derived from a metastatic site of pleural effusion. Similarly, although to a lower extent, the SI values of [C16Pyr][Amp] for HTB22 and HCC1937 were also remarkable. Compared to cisplatin, the SI value of [C16Pyr][Amp] was 8-fold higher in HTB22. Concerning the PCa cell lines, [C16Pyr][Amp] exhibited the greatest SI value in LNCaP (SI = 7.16), which is approximately 11-fold the value obtained for cisplatin. Importantly, in PC-3, this compound also presented an interesting SI (SI = 2.63), especially because of the metastatic and castration-resistant nature of this cell line. Hence, [C16Pyr][Amp] might be a potential compound for clinical purposes, with the highest selectivity for tumor cells without significantly impairing normal cell lines when compared to cisplatin.

### 2.3. [C16Pyr][Amp] Decreased Tumor Cell Viability and Increased Apoptosis Levels

**Figure 1:**
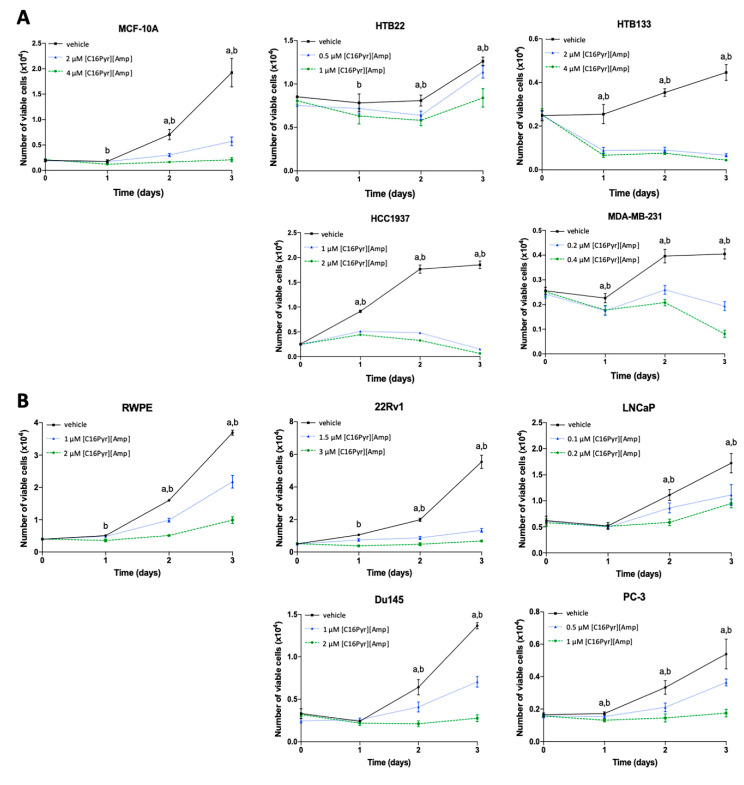
The impact of [C16Pyr][Amp] on cell viability at days 0, 1, 2, and 3 was measured by 3-(4,5-dimethylthiazol-2-yl)-2,5-diphenyltetrazolium-bromide (MTT) assay for the (**A**) breast (BrCa) and (**B**) prostate (PCa) cancer cell lines. Statistically significant differences (*p* < 0.05) were observed between (a) the vehicle and the lowest concentration, as well as (b) the vehicle and the highest concentration. All data are presented as the mean of three independent experiments ± SD.

Overall, a time- and dose-dependent reduction in the viability of BrCa and PCa cells was observed upon [C16Pyr][Amp] treatment (Figure 1). The decrease in tumor cell viability was evident from day 1, at least for the highest concentration, except for the PCa cell lines LNCaP and Du145. When exposed to [C16Pyr][Amp], the BrCa cell lines HTB133 and HCC1937 and the PCa cell line 22Rv1 were the most responsive, presenting the highest decrease in cell viability (76 to 96.5%) at both concentrations, contrarily to HTB22 (BrCa cell line) and LNCaP (PCa cell line), with only a 16–45% reduction. When comparing the cell viability of malignant cells with the corresponding benign cell lines, MCF-10A exhibited 3–4 times more viable cells compared to HCC1937 treated with both concentrations of [C16Pyr][Amp] and twice the number relative to HTB133 after treatment with 2 µM of [C16Pyr][Amp]. Likewise, RWPE also displayed a higher number of viable cells when compared to 22Rv1 upon exposure to both concentrations of [C16Pyr][Amp] (data not shown).

**Figure 2:**
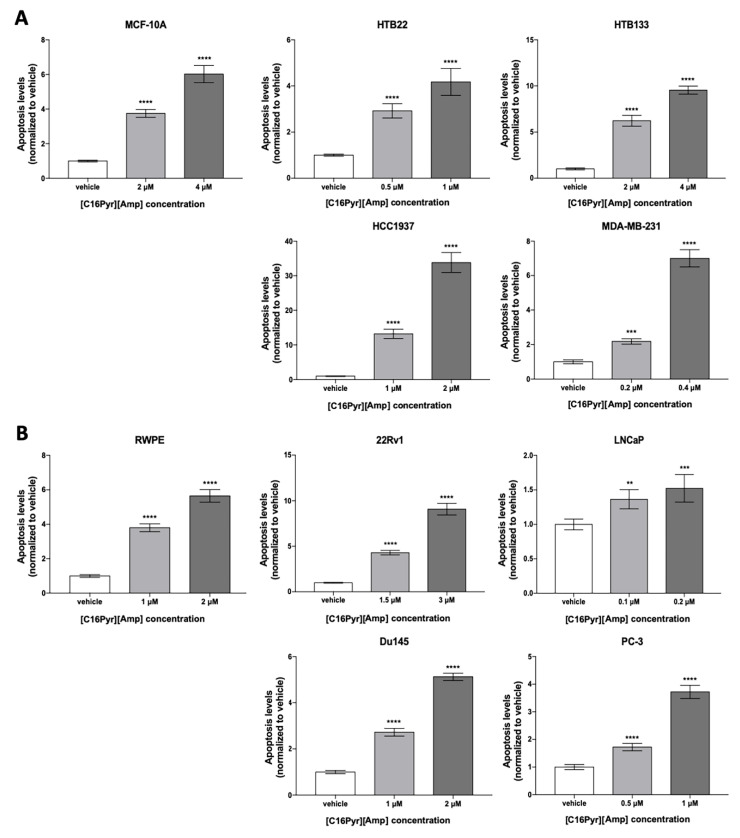
The impact of [C16Pyr][Amp] on apoptosis levels on day 3 after treatment was measured and compared with the vehicle for the (**A**) BrCa and (**B**) PCa cell lines. All data are presented as mean of three independent experiments ± SD. ** *p* < 0.01; *** *p* < 0.001; **** *p* < 0.0001).

These results were paralleled by those obtained in the analysis of the resistance to apoptosis. Indeed, [C16Pyr][Amp] significantly increased apoptotic levels in a dose-dependent manner in all cancer and benign cell lines (Figure 2). The HTB133, HCC1937, and 22Rv1 cell lines depicted up to a 34-fold increase in apoptosis levels after being treated with both doses of [C16Pyr][Amp]. These three cell lines also displayed the highest apoptosis levels when compared to their respective benign counterparts, with a 2–6-fold increase. Of note, the HCC1937 results were obtained with half of the [C16Pyr][Amp] concentration used for the breast benign cell line MCF-10A, showing a more exacerbated effect on malignant cell lines even with lower [C16Pyr][Amp] concentrations.

Regarding the cytotoxic effect, [C16Pyr][Amp] promoted a slight lactate dehydrogenase (LDH) increase in the medium (data not shown) comparative to the control condition, which is indicative of necrosis and cytotoxicity due to a damaged plasma membrane [41]. Herein, HTB22 and MDA-MB-231 (BrCa cell lines), as well as LNCaP and Du145 (PCa cell lines), showed the highest increase.

### 2.4. [C16Pyr][Amp] Reduced the Colony Formation Capacity of Tumor Cells

**Figure 3:**
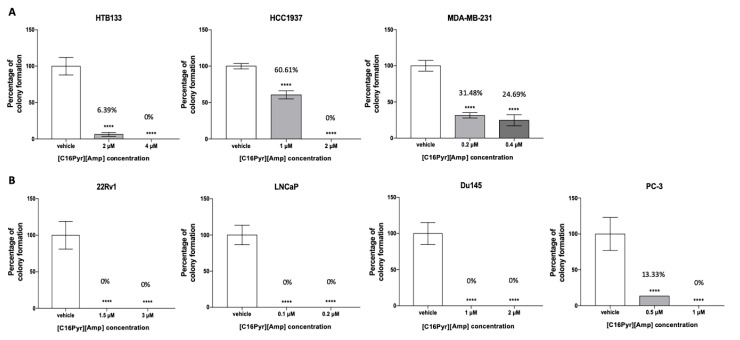
Effect of [C16Pyr][Amp] on the colony formation ability of the (**A**) BrCa and (**B**) PCa cell lines after treatment compared to the vehicle. All data are presented as mean of three independent experiments ± SD (**** *p* < 0.0001).

The in vitro colony formation assay is based on the ability of a single malignant cell to grow and divide, thereby forming colonies, contrarily to healthy cells [42]. All cell lines, except HTB22, were able to form colonies. After [C16Pyr][Amp] exposure, the fraction of surviving cancer cells was significantly reduced, and, consequently, the number of colonies formed was reduced in a dose-dependent manner (Figure 3). When exposed to [C16Pyr][Amp], the colony formation ability in the hormone-dependent cell lines displayed a higher inhibition than the hormone-independent BrCa and castration-resistant PCa cells at both concentrations, with a more pronounced effect on the PCa cell lines. Both BrCa cell lines HCC1937 and MDA-MB-231 showed a smaller decrease in the percentage of colony formation, mainly at the minor doses of 1 µM (60.61%) and 0.2 µM (31.48%), respectively. The BrCa cell line HTB22 was excluded from this assay due to its limited proliferative rate, reduced survival, growth capacity, and, consequently, low colony formation at a low cell concentration. In fact, this cell line was already shown to be the least responsive to [C16Pyr][Amp], according to the cell viability and apoptosis results.

### 2.5. CPT2, LDHA, MCM,2 and SKP2 Gene Expression Is Downregulated in Tumor Cells

Considering the antitumor activity of [C16Pyr][Amp] exhibited in the functional assays, the main cellular pathways possibly affected by [C16Pyr][Amp] treatment were assessed through the identification of altered gene expression that could corroborate the anticancer effects shown. For this purpose, the cell lines that best responded to treatment with [C16Pyr][Amp], identified in the previous trials and representative of the wide range of breast and prostate tumors, were chosen. Specifically, two hormone-dependent (HTB133 and 22Rv1) and two hormone-independent BrCa and castration-resistant PCa (MDA-MB-231 and Du145) cells were treated with the highest [C16Pyr][Amp] concentration and further tested via transcript analysis. A commercial custom array panel of cancer research was used to evaluate the expression of several genes involved in cell cycle, apoptosis, DNA repair, cellular metabolism, and mTOR or MAPK/ERK pathways. From the 96 genes analyzed in the array, four were downregulated in the four tested cell lines upon [C16Pyr][Amp] treatment: *CPT2*, *LDHA*, *MCM2,* and *SKP2* (Table 3 and Figure S4). The *CTP2* gene was significantly downregulated in both PCa cell lines, with a marked decrease in 22Rv1 (10.65-fold decrease, *p* = 0.006). Concerning the *MCM2* gene, a higher difference was depicted in HTB133 (2.78-fold decrease, *p* < 0.0001) and MDA-MB-231 (2-fold decrease, *p* = 0.003), although 22Rv1 also presented a statistically significant decrease. Both the *LDHA* and *SKP2* genes also depicted significantly decreased expression in the BrCa cell lines, *SKP2* being also significantly downregulated in both PCa cell lines, although to a lower extent. Curiously, the hormone-dependent cell lines (HTB133 and 22Rv1) depicted a more impressive decreased expression of these genes compared to the respective BrCa hormone-independent and PCa castration-resistant (MDA-MB-231 and Du145) cell lines.

## 3. Discussion

Despite technological and social development, cancer remains an important cause of morbidity and mortality. The global increment in cancer incidence and mortality rates, among several other complex reasons, is a consequence of demographic factors (e.g., aging and populational growth) and socioeconomic development, which alter the prevalence and distribution of cancer risk factors [1]. BrCa and PCa are some of the most common cancers worldwide [1], with a high rate of mortality mainly due to the aggressiveness of these tumors and their resistance to therapy [5,7]. Although there have been a lot of efforts in investigating new agents to improve cancer treatment and patients’ health, only around 10% of the drugs in clinical trials are launched in the market, highlighting the urgent need to increase this low successful percentage through the identification of new effective drugs [16,43]. Considering this scenario, the development of novel and more effective therapeutic agents is mandatory, with improved selectivity and less toxicity relative to conventional therapies.

ILs have been increasingly considered an important topic of investigation in the pharmaceutical industry, particularly concerning life sciences and medicine [14,17]. The third generation of ILs is a fusion of physical, chemical, and biological properties and provides improved water solubility and permeability, low toxicity, and better bioavailability [12,14]. The promising anticancer properties of these agents toward malignant cell lines of several tumor models have already been described [12,17,43,44,45,46]. The combination of ILs with chemotherapeutic agents has also been studied, with promising results in terms of reducing chemotherapy toxicity [47,48,49]. The high tunability of ILs implies their possible selectivity toward tumor cells without significantly impairing normal cells [12,14]. However, despite the investigation into the anticancer properties of ILs, there is still knowledge that needs to be explored and consolidated. In the case of [C16Pyr][Amp], there is only one study that, through IC_50_ determination, demonstrated that this compound has potent antiproliferative activity against five different human cancer cell lines, but with no exploration of the functional and genetic alterations of the cells treated. In that study, [C16Pyr][Amp] proved to be active in doses between 0.005 and 132.700 µM [12].

**Figure 4:**
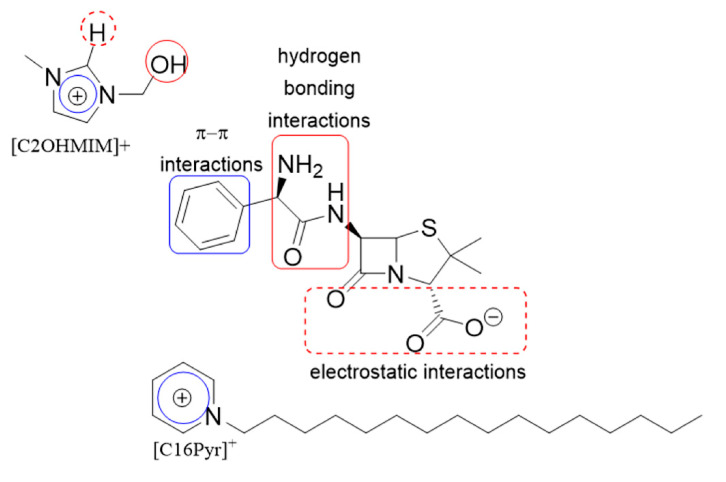
Structure of [C2OHMIM][Amp] and [C16Pyr][Amp] with their possible cation–anion interactions.

In our study, we evaluated the anticancer activity of two IL formulations containing the ampicillin anion, namely [C2OHMIM][Amp] and [C16Pyr][Amp], in BrCa and PCa cell lines. These compounds were previously reported to display a growth inhibitory effect in HTB133 and PC-3 cancer cells [12]. Although IC_50_ values of 0.146 and 0.297 µM were reported for HTB133 and PC-3, respectively, treated with [C2OHMIM][Amp][12], in this study, none of the tested cell lines responded to this compound. This might be explained by the different range of concentrations used to calculate the IC_50_ values, as well as the distinct growth medium and supplements to culture the cells, which can influence cell behavior and metabolism [50,51] and, consequently, the response to the treatment. Additionally, [C2OHMIM][Amp] was shown to be less cytotoxic than [C16Pyr][Amp] in the previously mentioned study. Conversely, lower IC_50_ values were obtained for [C16Pyr][Amp] in BrCa and PCa cell lines. These lower concentrations are possibly related to the hydrophobic nature of the [C16Pyr] cation conferred by the alkyl side chain [52]. ILs are organic salts that form a strong pair and it is the cation and the anion working together that are responsible for the activity [12]. This indicates that strong interaction between the cation and anion is very important. For example, the [C2OHMIM] anion can establish hydrogen bonding and π–π interactions and the [C16Pyr] anion can establish hydrogen bonding and π–π interaction but also has a long alkyl chain, as illustrated in Figure 4. The structure–activity relationship shows that ILs with long alkyl chains may be related to the high permeability of the membrane, altering the physical properties of the lipid bilayer. The long alkyl chains increase the lipophilic nature of the compounds, consequently increasing interactions with the phospholipid bilayer of the cell membrane and with the hydrophobic domains of membrane proteins, which may lead to the dissolution of the physiological functions of the membrane and ultimately lead to cell death [53]. Indeed, it has been described that toxicity increases with the length of the alkyl chain [52,54]. [C16Pyr][Amp] is also the compound with the highest inhibitory activity against cancer cells, without affecting normal fibroblasts [12]. Other ILs, such as those based on phosphonium and ammonium, have also been reported to have antitumor activity against a wide range of malignant cells [55].

Nonetheless, to the best of our knowledge, we are the first to report the role of [C16Pyr][Amp] formulation in inhibiting or attenuating the aggressive features of cancer cells. Overall, this compound was effective in attenuating the malignant phenotype of cancer cells by reducing cell viability and colony formation ability while inducing apoptosis. Moreover, the SI of [C16Pyr][Amp] revealed selectivity toward the majority of the tested tumor cells, mainly for MDA-MB-231 (SI = 11.00) and LNCaP (SI = 7.16), contrarily to the cisplatin SI values. This is a rather promising result, mainly for MDA-MB-231, since it is an aggressive cell line derived from a metastasis of pleural effusion. Moreover, [C16Pyr][Amp] presented a 2–11-fold augmentation of the SI values compared to cisplatin, a drug commonly used in the clinic, thus suggesting the higher selectivity of [C16Pyr][Amp] than cisplatin. In general, cancer is mainly treated with long-term intensive chemotherapy sessions to shrink the tumor so that it can effectively be removed by surgery, if necessary. However, chemotherapy is a highly debilitating therapeutic approach for patients, with severe side effects due to the lack of selectivity toward tumor cells. Despite its cytotoxicity, cisplatin is a widely known chemotherapeutic agent frequently used in the clinic, as well as docetaxel, doxorubicin, cyclophosphamide, and paclitaxel, already reported in BrCa and PCa cell lines (Table S1). In order to reduce chemotherapy toxicity, recently, a paclitaxel formulation based on ILs with promising results has been reported. Briefly, paclitaxel solubility and stability were improved, and less cytotoxicity and reduced hypersensitive reactions were exhibited compared to paclitaxel alone [47]. Hence, the combination of chemotherapeutic drugs with [C16Pyr][Amp] might be interesting to further investigate.

The MTT assay is among the most commonly used methods to evaluate the cytotoxicity of a chemical through the analysis of the capacity of mitochondrial enzymes to reduce the tetrazolium dye MTT to formazan crystals [14,56]. However, a cytotoxicity assay in molecular biology classically assesses cell death by the level of damage of the plasma membrane of a cell population. Moreover, LDH is a stable cytoplasmic enzyme, present in nearly all eukaryotic living cells, which is rapidly released into the cell culture medium upon damage of the plasma membrane [46,57,58]. Based on this, our study assessed both effects to better infer the possible mechanism of action of [C16Pyr][Amp]. Considering the MTT assay, all cell lines displayed a significant dose- and time-dependent decrease in cell viability upon exposure to [C16Pyr][Amp]. An exacerbated effect was achieved in two BrCa cell lines, i.e., HTB133 and HCC1937, and the PCa cell line 22Rv1 at both concentrations. These cell lines also depicted the highest dose-dependent apoptosis levels, and no increased LDH was found in the supernatant (data not shown) compared to the control condition. Therefore, these data indicate that [C16Pyr][Amp] might induce apoptosis, a programmed and controlled cell death mechanism. Conversely, the BrCa cell lines HTB22 and MDA-MB-231 and the PCa cell lines LNCaP and Du145 presented lower apoptosis but increased LDH levels, although to a lower extent (data not shown). The permeabilization of the plasma membrane compromises its integrity, being a key signature of necrotic cells [41]. Contrarily to apoptosis, the recruitment of immune cells in the neoplasia context by necrotic cells has a pro-inflammatory effect and actively contributes to tumor promotion [59]. However, since this LDH increase was very low, and because these cell lines also depicted apoptosis levels in accordance with decreased cell viability, this suggests that the cell death mechanism implied in these cells does not boost tumorigenesis. PC-3 was the less affected cell line in terms of treatment with C16: although cell viability was decreased, the apoptosis levels were not exacerbated compared to the other cell lines and did not present increased LDH levels in the culture medium. Comparing the PCa cell lines treated with the same concentrations, 1 µM of [C16Pyr][Amp] showed a more pronounced cell viability reduction and an increase in apoptosis in Du145 and PC-3 cells compared to those obtained for the benign prostate cell line RWPE. The same was found for 2 µM of [C16Pyr][Amp], where the BrCa cell lines HTB133 and HCC1937 displayed a higher cell viability reduction and apoptosis augmentation compared to the benign breast cell line MCF-10A. These results suggest that [C16Pyr][Amp] at 1 and 2 µM is effective in decreasing the tumorigenic features of cancer cells without significantly impairing benign cell lines. Moreover, [C16Pyr][Amp] was also shown to be more selective for cancer cells than the well-known chemotherapeutic agent cisplatin.

Kaushik et al. reported that ammonium and imidazolium ILs also inhibit the colony formation capability of brain cancer cells in a concentration-dependent manner [60]. Herein, [C16Pyr][Amp] also reduced the colony formation ability of BrCa and PCa cell lines in a dose-dependent manner. Specifically, in both tumor models, the effect was less apparent in the cell lines with a higher colony forming ability. Contrary to the results in previous assays, the most significant effect was found in the PCa cell lines. Altogether, the functional assays revealed that [C16Pyr][Amp] efficiently reduced cell viability and colony formation, while it induced the cell death of all BrCa and PCa cell lines, not being specific for a tumor model or tumor phenotype.

In order to dissect the possible molecular pathways and mechanisms involved in the effects of [C16Pyr][Amp], a gene expression panel of 96 genes was analyzed and revealed downregulation of the *CPT2*, *LDHA*, *MCM2,* and *SKP2* genes upon treatment with [C16Pyr][Amp]. These genes are mainly involved in cellular metabolism and cell division and their decreased expression corroborates the exacerbated effects observed on cell viability and apoptosis after [C16Pyr][Amp] exposure. *CPT2* encodes a mitochondrial enzyme involved in lipid metabolism, regulating fatty acid oxidation (FAO) in normal cells [61]. In cancer cells, the most advantageous fuels are fatty acids, since mitochondrial FAO produces a higher amount of ATP than the oxidation of glucose or amino acids, highlighting its crucial role in the energy homeostasis of cancer cell metabolism [62]. The strong dependency in mitochondrial FAO induces resistance to nutrient deprivation and environmental stress inducers in some tumors [63]. Indeed, even under abundant nutrient conditions, PCa cells promote FAO as the main source of energy production and express high levels of FAO enzymes [64]. [C16Pyr][Amp] treatment of the PCa cells induced a pronounced decrease in *CPT2* expression, mainly in the 22Rv1 cell line, indicating that this IL might shut down the most profitable cellular energy source of malignant cells. Another important energy source of cancer cells is the oxidation of glucose during glycolysis with lactate production at high levels to support rapid cell growth, supplying metabolic intermediates for macromolecule biosynthesis [65]. In cancer cells, a large fraction of pyruvate is converted into lactate preferentially by LDHA, with NAD^+^ as a cofactor, even with high oxygen availability (called the Warburg effect), minimizing pyruvate’s entry into the Krebs cycle in the mitochondria [66]. LDHA is commonly upregulated in several rapidly grown tumors [67,68,69,70,71], allowing cancer cells to survive and proliferate under hypoxic conditions (0.5% oxygen) [66,72]. LDHA phosphorylation elicits its activation on BrCa cells, promoting invasion and metastasis with enhanced anoikis resistance [70]. Indeed, LDHA knockout in xenograft BrCa cell lines has been shown to increase the levels of pro-apoptotic proteins and to reduce Bcl-2 expression [73]. In the same line, [C16Pyr][Amp] treatment induces elevated apoptosis levels that might be explained by the statistically significant reduction of *LDHA* expression in BrCa cells, reflecting the phenotype of the molecular effect found in the xenograft study. Interestingly, LDHA targeting has been reported to sensitize cancer cells to the cytotoxic effects of chemotherapy [74], including paclitaxel-resistant BrCa cells [75]. Therefore, and as aforementioned, the combination of [C16Pyr][Amp] with cisplatin or another chemotherapeutic drug should be considered in the future. Overall, our data suggest that [C16Pyr][Amp] impairs the hallmark of cancer related to the reprogramming of cellular energy and metabolism [76], decreasing both *CPT2* (in PCa cell lines) and *LDHA* (in BrCa cell lines) expression, which affects the energy supply by FAO and the lactate production during glycolysis, respectively. Consequently, the tumorigenic potential of cancer cells is diminished.

[C16Pyr][Amp] treatment of the BrCa and PCa cell lines also significantly downregulated *MCM2* and *SKP2* expression implicated in eukaryotic DNA replication and cell cycle regulation, respectively. Indeed, in normal cell lines, the correct initiation of DNA replication is fundamental to maintaining genomic integrity and stability. The efficiency of this biological process is dependent on the formation of pre-replicative complexes in the late M/early G phase through the recruitment of MCM2-7 molecules. Upon MCM phosphorylation, the replicative helicase complex is formed with robust helicase activity to initiate DNA replication and unwinding [77,78]. MCM2 was previously shown to be overexpressed in BrCa cells and associated with poor prognosis and therapy resistance [79,80]. Moreover, in PCa, MCM2 immunohistochemistry expression is associated with short-term survival [81] and predicts biochemical recurrence [82]. Herein, decreased *MCM2* mRNA upon [C16Pyr][Amp] exposure paralleled the reduced cell proliferation found in in vitro assays. Indeed, MCM2 has been proposed to be an alternative proliferation marker to ki67 in the BrCa model [83]. Both MCM2 and SKP2 are implicated in the G_1_/S phase transition of the mitotic cell cycle and are reported to be co-expressed in lung and squamous cell carcinoma tissue samples [84], showing the close relationship between these two proteins. *SKP2* is an oncogene that encodes a protein that regulates cell cycle entry and G_1_/S transition through a negative feedback loop targeting p21 and p27 degradation, thereby inhibiting cyclin-dependent kinases [85,86]. SKP2 overexpression has been already described in BrCa and PCa, contributing to the development and proliferation of these tumors, the acquisition of a mesenchymal phenotype, and the resistance to radio- and chemotherapy [87,88,89,90], being a potential therapeutic target [73]. Accordingly, [C16Pyr][Amp] showed promising results by reducing the *SKP2* mRNA levels in the BrCa and PCa cell lines. Contrarily, *SKP2* depletion is associated with the diminished disease progression, epithelial-to-mesenchymal transition, and stem cell features of castration-resistant PCa in a twist destabilization-dependent mechanism [91]. Hence, [C16Pyr][Amp] treatment might also have an inhibitory effect on the epithelial-to-mesenchymal transition capacity through *SKP2* downregulation. In BrCa, SKP2 expression downregulation by doxorubicin has been found to induce cell cycle arrest in the G_1_/M or G_2_/M phase in BrCa cell lines, with differential effects depending on the cell cycle checkpoint activated [92]. In the same line, in our study, the *MCM2* and *SKP2* downregulation upon [C16Pyr][Amp] treatment suggests a role of this IL in DNA replication and cell cycle rate reduction in both BrCa and PCa cell lines.

Our study demonstrated that [C16Pyr][Amp] has potential as an anticancer therapeutic agent in BrCa and PCa cell lines with disparate phenotypes. The tumorigenic features of these cell lines were impaired upon [C16Pyr][Amp] treatment, leading to a decreased number of malignant viable cells, accompanied by increased cell death, mainly by apoptosis. The aggressiveness of malignant cells was also quantitatively diminished, herein reflected by a reduction in colony formation capacity. At the molecular level, downregulation of the genes involved in two well-known hallmarks of cancer, namely cellular energetics and sustaining the proliferative signaling in the context of DNA replication and cell cycle regulation, might contribute to the malignant behavior attenuation of cell lines upon [C16Pyr][Amp] treatment in phenotypic assays. The selectivity and lower toxicity of [C16Pyr][Amp] compared to cisplatin render it a promising therapeutic agent that might be administered alone or in combination with the currently used conventional therapies. In vivo studies are needed to further evaluate the antitumor effect of C16, as well as to identify targetable proteins that may serve as surrogate markers of therapy response in BrCa and PCa patients.

## 4. Materials and Methods

### 4.1. Cell Culture

Ten American Type Culture Collection (ATCC) (Lockville, MD, USA) immortalized epithelial cell lines available at the Instituto Português de Oncologia do Porto laboratory were cultured in the recommended medium supplemented with 10% fetal bovine serum (FBS) (Merck, Berlin, Germany) and a 1% antibiotic–antimycotic (Anti–Anti (100×), Gibco, Waltham, MA, USA). The cancer cell lines chosen present distinct features, covering a large spectrum of breast and prostate tumors, while the benign cell lines were used as controls. Briefly, minimum essential media (MEM) was used to culture the HTB22 and Du145 cell lines; Roswell Park Memorial Institute (RPMI)-1640 was used to culture the HTB133, HCC1937, 22Rv1, and LNCaP cell lines; Dulbecco’s Modified Eagle Medium (DMEM) was used to culture the MDA-MB-231 cell line; RPMI-1640/Ham’s F-12 Nutrient Mixture (50:50, *v/v*) was used to culture the PC-3 cell line; Keratinocyte Serum-Free Medium (SFM) was used to culture the RWPE-1 cell line (Gibco). Specifically, MCF-10A was cultured with DMEM/F12 (50:50, *v/v*) supplemented with 5% horse serum (Gibco), 20 ng/mL of Epidermal Growth Factor (EGF) (PeproTech, London, UK), 0.5 mg/mL of hydrocortisone (Sigma-Aldrich, St. Louis, MO, USA), 100 ng/mL of cholera toxin (Sigma-Aldrich), 10 µg/mL of insulin (Sigma-Aldrich), and 1% penicillin–streptomycin (Gibco). All cells were maintained at 37 °C in a humidified atmosphere containing 5% CO_2_ and routinely tested for *Mycoplasma* spp. contamination (PCR Mycoplasma Detection Set, Takara Bio, Shiga, Japan).

### 4.2. Compound Formulation

Four synthetic compounds were used in this study, including two quinoxalines (quinoxaline-1,4-dioxide and 2-methylquinoxalinep1,4-dioxide) and two ILs ([C16Pyr][Amp] (C16) and [C2OHMIM][Amp]) based on ampicillin. The quinoxaline derivates were purified by reduced pressure sublimation, and thermal stability was verified by differential scanning calorimetry (DSC), as previously described [93,94]. The ILs based on ampicillin were prepared by an optimized and sustainable buffer neutralization method, as described by Ferraz et al. [95]. Synthesis and spectral data of [C16Pyr][Amp] and [C2OHMIM][Amp] are supplied as Supplementary Materials. The quinoxalines and ILs were dissolved in purified water and dimethyl sulfoxide (DMSO) (Sigma-Aldrich), respectively, to concentrations from 500 µM to 0.5 nM in serial dilutions of 1:10. Work solutions with a solvent final concentration of 1% were prepared for all of the tested concentrations to avoid solvent effects during treatment.

### 4.3. Cell Viability Assay, IC_50_, and Selectivity Index Assessment

The IC_50_ value and the dose–response curves of each compound were determined with an MTT assay. Cells were seeded onto 96-well flat-bottomed culture plates at 2 × 10^3^ (MCF-10A and Du145), 7 × 10^3^ (HTB22), 2.5 × 10^3^ (HTB133, HCC1937, MDA-MB-231, and RWPE), 4 × 10^3^ (22Rv1), 5 × 10^3^ (LNCaP), and 1.6 × 10^3^ (PC-3) cells per well. The IC_50_ value was determined using nonlinear regression (curve fit) with all logarithmic absorbance values. For control purposes, cell lines were also exposed to the compounds’ vehicles, i.e., purified water and DMSO for the quinoxalines and ILs, respectively. To assess the toxicity of the compounds, cisplatin was used as a control. All cells were treated 24 h after being cultured (day 0) and were evaluated with 72 h of exposure to the compounds (day 3). The IC_50_ values of cisplatin were calculated for all cell lines after 72 h with concentrations of 50 nM, 500 nM, 5 µM, 50 µM, and 100 µM, considering the IC_50_ values previously described in the literature [96,97,98,99,100,101]. For control purposes, cells were treated with cisplatin solvent (saline solution). Cisplatin was always freshly prepared immediately before use.

For each compound, the selectivity for the tumor cells relative to the normal ones was assessed by calculating the SI, i.e., the ratio between the IC_50_ values of the normal and tumor cell lines. The higher the SI value, the greater the selectivity of the compound toward the tumor cells. Indeed, a compound with SI > 3 is highly selective for tumor cells [102].

Cell viability was assessed using an MTT assay (Sigma-Aldrich), as previously described [103]. Cells were allowed to adhere overnight and then exposed to two different compound concentrations, i.e., the IC_50_ concentration and one concentration above once. MTT (0.5 mg/mL) was added to each well and the viability measured every day until 72 h using a microplate reader (FLUOstar Omega, BMG Labtech, Offenburg, Germany) at a wavelength of 540 nm with background subtraction at 630 nm. Three replicates were used for each condition and at least three biological independent experiments were performed. The number of viable cells was calculated as follows: (experiment Optical Density (OD) × number of cells at day 0)/mean OD at day 0.

### 4.4. Apoptosis Assay

Apoptosis was evaluated using the APOPercentage^TM^ apoptosis assay kit (Biocolor Ltd., Belfast, Northern Ireland), according to the manufacturer’s instructions. Briefly, 4 × 10^3^ (MCF-10A), 3.25 × 10^4^ (HTB22), 1 × 10^4^ (HTB133), 4.5 × 10^3^ (HCC1937), 3.5 × 10^3^ (MDA-MB-231), 6.25 × 10^3^ (RWPE), 2.25 × 10^4^ (22Rv1), 1.5 × 10^4^ (LNCaP), 5 × 10^3^ (Du145), and 3 × 10^3^ (PC-3) cells were seeded per well onto 24-well plates and allowed to adhere overnight. At 72 h, 5% of the kit dye was added to the media and incubated at 37 °C for 15–30 min. As a positive control, cells were treated with hydrogen peroxide. After exposure to a dye releasing agent, the absorbance was determined in a microplate reader (FLUOstar Omega) at a wavelength of 550 nm with background subtraction at 620 nm. Three biological and three experimental replicates were performed for each condition. Apoptosis levels were calculated using the following formula: Apoptosis OD/mean MTT OD at day 3.

### 4.5. Cytotoxicity Assay

The cytotoxic effect of the compounds was evaluated using a commercial lactate dehydrogenase (LDH) kit (Pyruvate–Kinetic–UV Kit, SPINREACT, Barcelona, Spain), following the manufacturer’s recommendations. Briefly, this kit directly measured the NADH concentration in the cell-conditioned media, which is inversely proportional to the LDH activity; a lower NADH concentration is associated with more lysed cells, suggesting cytotoxicity of the compound used. NADH concentration was photometrically measured at 340 nm and the data were analyzed with the following formula: (vehicle-treated)/vehicle × 100.

### 4.6. Colony Formation Assay

Both the BrCa and PCa cell lines were seeded in 6-well culture plates at specific concentrations: 7.5 × 10^2^ cells/mL for HTB133, MDA-MB-231, 22Rv1, and Du145; 3.75 × 10^2^ cells/mL for LNCaP; 2.5 × 10^2^ cells/mL for HCC1937; 1.0 × 10^2^ cells/mL for PC-3. Cells were allowed to adhere overnight and then treated. Since the time needed for colony formation was more than 72 h and varied for each cell line (5–6 days for HCC1937, MDA-MB-231, Du145, and PC-3; 10–12 days for HTB133 and 22Rv1; 18 days for LNCaP), the treatment was repeated every 3 days. Colonies were stained with 25% (*w/v*) Giemsa in dH_2_O. Colonies were defined with at least 50 cells each, as done previously [104], and counted using an Olympus IX51 microscope (Olympus, Tokyo, Japan). The data were analyzed following the formula: (Colony number/control group colony number) × 100.

### 4.7. RNA Quantification, Reverse Transcription, and Real-Time PCR

After compound exposure, RNA was extracted from the cell lines using TRIzol^®^ (Invitrogen, Carlsbad, CA, USA), according to manufacturer’s instructions. The extracted RNA was quantified using a Nanodrop Life Spectrophotometer (Nanodrop Technologies, Wilmington, DE, USA). Furthermore, 400 ng of complementary DNA (cDNA) was synthetized using a Transcriptor High Fidelity cDNA Synthesis Kit (Roche, Basel, Switzerland), according to manufacturer’s instructions. The RT^2^ Profiler PCR Array System Kit (Qiagen, Hilden, Germany) included 96 genes corresponding to cancer research molecular pathways and adequate controls in quadruplicate. The expression levels were determined by real-time PCR in a LightCycler 480 (Roche Diagnostics) and *ACTINB*, *GAPDH*, and *HPRT1* were used as endogenous controls. The RT^2^ profiler PCR array analysis was performed using the Qiagen-specific platform. The data analysis in the web portal calculated fold change using the ΔΔCT method. Genes with a logarithmized fold change above 1 or below −1 were considered. Additionally, the DNA genomic contamination (GDC), as well as the first strand synthesis (RTC) and real-time PCR efficiency (PPC), were monitored using the Qiagen platform for the RT^2^ profiler PCR array analysis. The lower limit of detection was set at CT ≥ 35.

### 4.8. Statistical Analysis

One-way analysis of variance (ANOVA) with the post-hoc Dunnett’s multiple comparison test was used to compare the results obtained in each parameter for the different compounds concentrations and the control/vehicle, when appropriate. Comparison of IC_50_ and SI values of [C16Pyr][Amp] and cisplatin was carried out using Wilcoxon matched pair tests and Pearson’s correlations. For RNA expression in the RT^2^ profiler PCR array, *p*-values were calculated based on a Student’s *t*-test of the replicate 2^(−ΔΔCT)^ values for each gene in the control and treatment groups. Analysis was performed with GraphPad Prism 7, and statistical significance was set at *p* < 0.05.

## Figures and Tables

**Table 1:** Half-maximal inhibitory concentration (IC_50_) values of [C16Pyr][Amp] and cisplatin for different breast and prostate cell lines. All data are presented as mean of three independent experiments ± standard deviation (SD).

Model	Cell Lines	IC_50_ Value ± SD (µM)	
		[C16Pyr][Amp]	Cisplatin
Breast	MCF-10A	2.1 ± 0.27	4.9 ± 0.42
	HTB22	0.6 ± 0.08	10.9 ± 0.62
	HTB133	2.0 ± 0.55	8.2 ± 0.69
	HCC1937	0.8 ± 0.01	3.5 ± 0.25
	MDA-MB-231	0.2 ± 0.01	1.1 ± 0.10
Prostate	RWPE	0.9 ± 0.13	4.9 ± 0.06
	22Rv1	1.4 ± 0.16	5.5 ± 0.18
	LNCaP	0.1 ± 0.02	7.4 ± 0.33
	Du145	0.9 ± 0.08	0.6 ± 0.01
	PC-3	0.3 ± 0.00	5.1 ± 0.67

**Table 2:** Selectivity index (SI) of [C16Pyr][Amp] and cisplatin for each breast (BrCa) and prostate (PCa) cancer cell line.

Tumor Model	Cell Lines	Selectivity Index	
		[C16Pyr][Amp]	Cisplatin
Breast cancer	HTB22	3.73	0.45
	HTB133	1.05	0.60
	HCC1937	2.66	1.41
	MDA-MB-231	11.00	4.37
Prostate cancer	22Rv1	0.66	0.89
	LNCaP	7.16	0.66
	Du145	1.01	8.20
	PC-3	2.63	0.96

**Table 3:** *CPT2*, *LDHA*, *MCM2,* and *SPK2* fold change values between the vehicle and the [C16Pyr][Amp] treatment conditions for the selected BrCa and PCa cell lines.

Genes	HTB133		MDA-MB-231		22Rv1		Du145	
	Fold Change	*p*-Value	Fold Change	*p*-Value	Fold Change	*p*-Value	Fold Change	*p*-Value
*CPT2*	−1.51	0.160	−1.22	0.360	−10.65	0.006	−1.73	0.004
*LDHA*	−5.21	<0.001	−3.68	0.0001	−1.04	0.840	−1.15	0.203
*MCM2*	−2.78	<0.0001	−1.99	0.003	−1.55	0.030	−1.18	0.163
*SKP2*	−2.90	<0.0001	−2.86	<0.0001	−2.36	0.030	−1.96	0.002

## Supplementary Materials

Supplementary materials can be found at https://www.mdpi.com/1422-0067/21/24/9584/s1. Figure S1. Chemical structure of quinoxaline-1,4-dioxide, 2-methylquinoxaline-1,4-dioxide, [C2OHMIM][Amp], and [C16Pyr][Amp]; Figure S2. Dose–response curves of (a) quinoxaline-1,4-dioxide, (b) 2-methylquinoxaline-1,4-dioxide, (c) [C16Pyr][Amp], and (d) [C2OHMIM][Amp] in (A) breast (BrCa) and (B) prostate (PCa) cancer cell lines using nonlinear regression (curve fit) with all logarithmic absorbance values. All data are presented as mean of three independent experiments ± standard deviation (SD); Figure S3. Dose–response curves of cisplatin in (A) BrCa and (B) PCa cell lines using nonlinear regression (curve fit) with all logarithmic absorbance values. All data are presented as mean of three independent experiments ± SD; Figure S4. Gene expression array of cancer research molecular pathways for the (A) HTB133, (B) MDA-MB-231, (C) 22Rv1, and (D) Du145 cell lines. Abbreviations: C16, [C16Pyr][Amp]. Table S1. Half-maximal inhibitory concentration (IC50) values of docetaxel, doxorubicin, cyclophosphamide, and paclitaxel cisplatin for different breast and prostate cell lines with indication of the duration and assay used.

## Abbreviations

ANOVA	One-way analysis of variance
BrCa	Breast cancer
cDNA	Complementary DNA
DMEM	Dulbecco’s Modified Eagle Medium
DMSO	Dimethyl Sulfoxide
DSC	Differential scanning calorimetry
EGF	Epidermal Growth Factor
FAO	Fatty acid oxidation
FBS	Fetal bovine serum
GDC	DNA genomic contamination
IC_50_	Half-maximal inhibitory concentration
IL	Ionic liquid
LDH	Lactate dehydrogenase
MEM	Minimum essential media
MTT	3-(4,5-dimethylthiazol-2-yl)-2,5-diphenyltetrazolium-bromide
OD	Optical Density
PCa	Prostate cancer
PPC	Real-time PCR efficiency
RTC	First strand synthesis
SFM	Serum-Free Medium
SI	Selectivity index
