# Supplementary material for: The Impact of [C16Pyr][Amp] on the Aggressiveness in Breast and Prostate Cancer Cell Lines
Source: Int J Mol Sci. 2020 Dec 16;21(24):9584. doi: 10.3390/ijms21249584 (PMC7765672; doi:10.3390/ijms21249584)
Supplement: Supplementary file 1 [file ijms-21-09584-s001.zip › Suplemmentary/ijms-954203_supplementary.pdf]

International Journal of *Molecular Sciences*

MDPI

Article

The Impact of [C16Pyr][Amp] on the Aggressiveness in Breast and Prostate Cancer Cell Lines

Filipa Quintela Vieira [1,2,3,†], Ângela Marques-Magalhães [1,3,†], Vera Miranda-Gonçalves [3], Ricardo Ferraz [1,4,5], Mónica Vieira [1,5,6], Cristina Prudêncio [1,5,6], Carmen Jerónimo [3,7,*,‡] and Regina Augusta Silva [1,2,*,‡]

[1] Research Centre in Health and Environment (CISA), School of Health (ESS), Polytechnic Institute of Porto (P.PORTO), 4200-072 Porto, Portugal; afv@eu.ipp.pt (F.Q.V.); magalhaes.angela94@gmail.com (Â.M.-M.); rferraz@ess.ipp.pt (R.F.); mav@ess.ipp.pt (M.V.); cprudencio@ess.ipp.pt (C.P.)
[2] Department of Pathological, Cytological and Thanatological Anatomy, ESS|P.PORTO, 4200-072 Porto, Portugal
[3] Cancer Biology and Epigenetics Group, IPO Porto Research Center (CI-IPOP), Portuguese Oncology Institute of Porto (IPO Porto), 4200-072 Porto, Portugal; Vera.Miranda.Goncalves@ipoporto.min-saude.pt
[4] LAQV-REQUIMTE, Departamento de Química e Bioquímica, Faculdade de Ciências, Universidade do Porto, 4169-007 Porto, Portugal
[5] Ciências Químicas e das Biomoléculas, ESS|P.PORTO, 4200-072 Porto, Portugal
[6] Instituto de Investigação e Inovação em Saúde (i3S), Universidade do Porto, 4200-072 Porto, Portugal
[7] Department of Pathology and Molecular Immunology, Institute of Biomedical Sciences Abel Salazar (ICBAS), University of Porto, 4050-313 Porto, Portugal
* Correspondence: carmenjeronimo@ipoporto.min-saude.pt or cljeronimo@icbas.up.pt (C.J.); ras@eu.ipp.pt (R.A.S.)
† These authors contributed equally to this work.
‡ Joint senior authors.

Received: 18 September 2020; Accepted: 14 December 2020; Published: 16 December 2020

Supplementary Materials

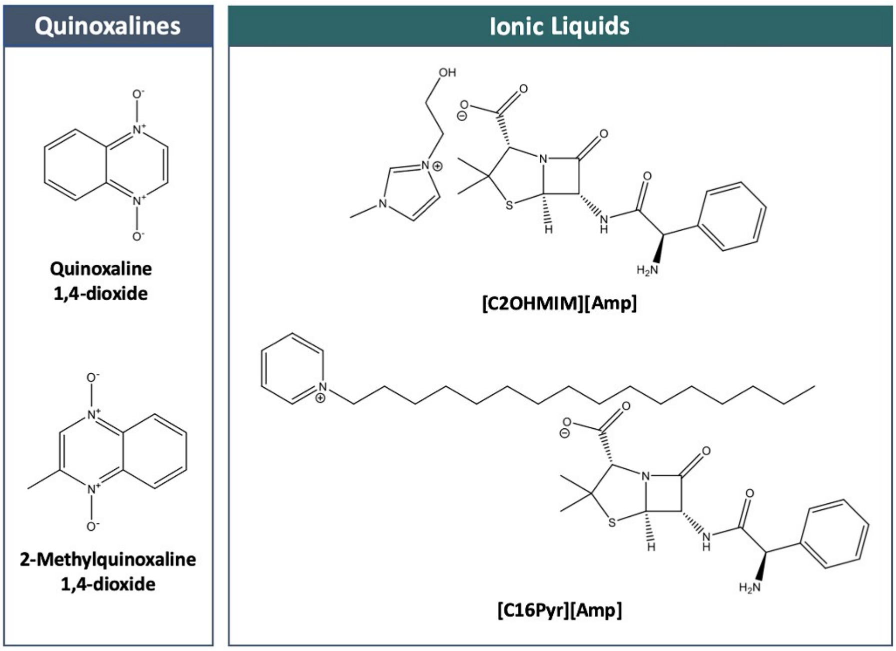

Figure S1 – Chemical structure of quinoxaline-1,4-dioxide, 2-methylquinoxaline-1,4-dioxide, [C2OHMIM][Amp], and [C16Pyr][Amp].

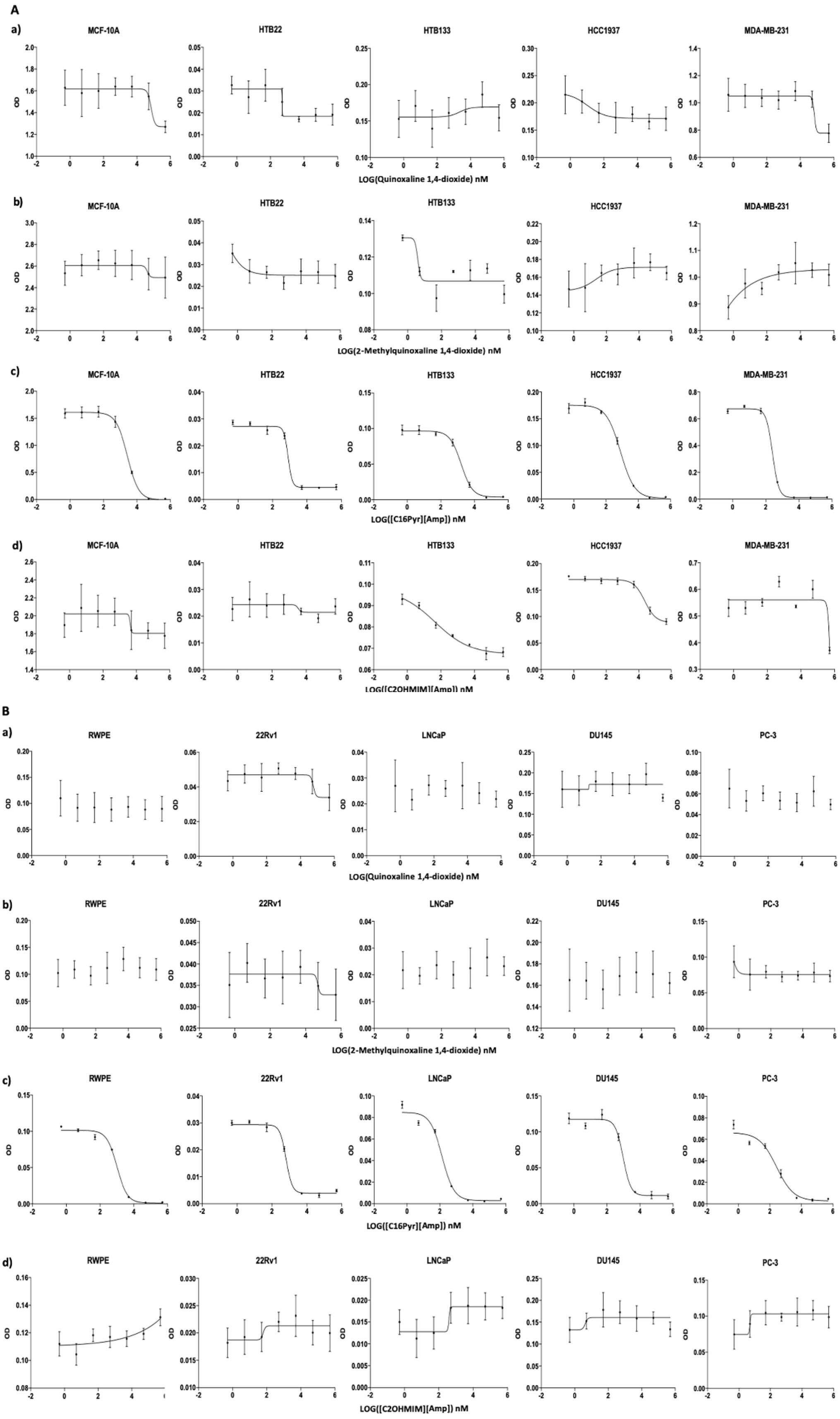

Figure S2 – Dose–response curves of (a) quinoxaline-1,4-dioxide, (b) 2-methylquinoxaline-1,4-dioxide, (c) [C16Pyr][Amp], and (d) [C2OHMIM][Amp] in (A) breast (BrCa) and (B) prostate (PCa) cancer cell lines using nonlinear regression (curve fit) with all logarithmic absorbance values. All data are presented as mean of three independent experiments standard deviation (SD).

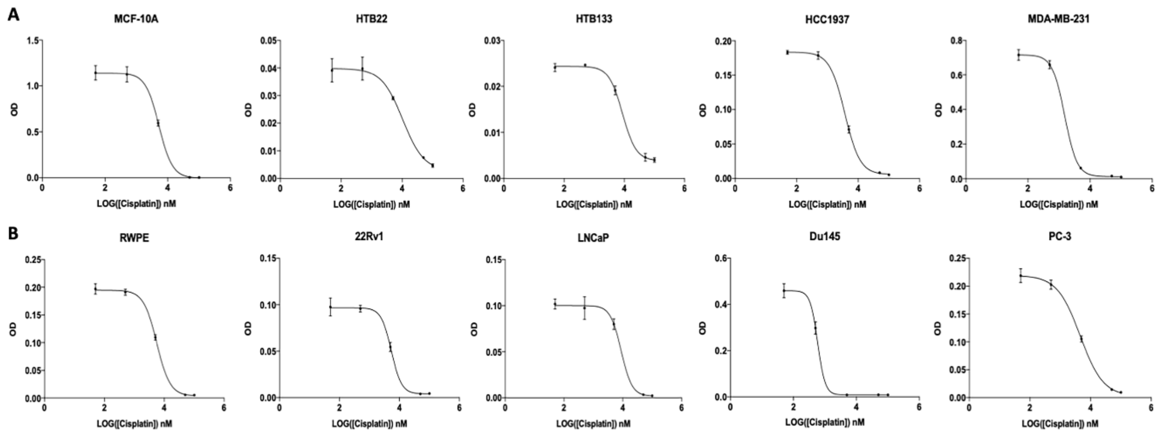

Figure S3 – Dose–response curves of cisplatin in (A) BrCa and (B) PCa cell lines using nonlinear regression (curve fit) with all logarithmic absorbance values. All data are presented as mean of three independent experiments ± SD;

A

HTB133

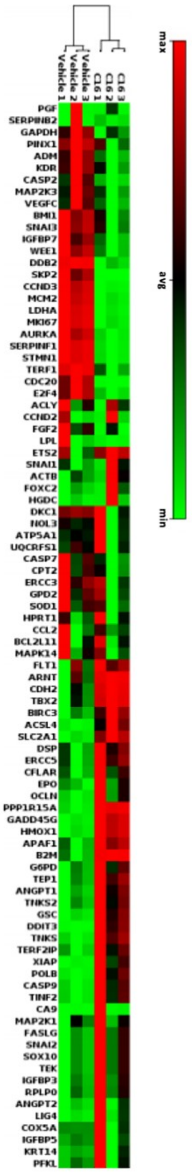

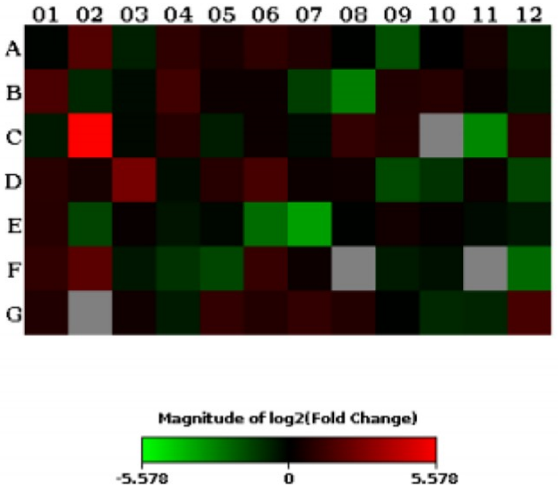

Layout	01	02	03	04	05	06	07	08	09	10	11	12
A	ACLY<br>-1.06	ACSL4<br>3.42	ADM<br>-1.59	ANGPT1<br>2.01	ANGPT2<br>1.42	APAF1<br>2.01	ARNT<br>1.62	ATP5A1<br>-1.04	AURKA<br>-3.38	BCL2L11<br>-1.00	BIRC3<br>1.38	BMI1<br>-1.73
B	CA9<br>3.07<br>B	CASP2<br>-1.79	CASP7<br>-1.17	CASP9<br>2.67	CCL2<br>1.22<br>B	CCND2<br>1.21<br>B	CCND3<br>-2.53	CDC20<br>-6.67	CDH2<br>1.64	CFLAR<br>1.77	COX5A<br>1.17	CPT2<br>-1.51
C	DDB2<br>-1.44	DDIT3<br>47.78	DKC1<br>-1.12	DSP<br>1.79	E2F4<br>-1.52	EPO<br>1.21<br>B	ERCC3<br>-1.17	ERCC5<br>2.13	ETS2<br>1.73	FASLG<br>1.28<br>C	FGF2<br>-7.93<br>B	FLT1<br>1.94
D	FOXC2<br>1.86<br>B	G6PD<br>1.38	GADD45<br>G<br>5.97	GPD2<br>-1.19	GSC<br>1.78	HMOX1<br>2.89	IGFBP3<br>1.19	IGFBP5<br>1.23	IGFBP7<br>-3.07	KDR<br>-2.14	KRT14<br>1.20<br>B	LDHA<br>-2.78
E	LIG4<br>1.80	LPL<br>-2.77<br>B	MAP2K1<br>1.13	MAP2K3<br>-1.36	MAPK14<br>-1.10	MCM2<br>-5.21	MKI67<br>-11.45	NOL3<br>-1.04	OCLN<br>1.31	PFKL<br>1.09	PGF<br>-1.15<br>B	PINX1<br>-1.40
F	POLB<br>2.05	PPP1R15A<br>3.91	SERPINB2<br>-1.40<br>B	SERPINF1<br>-2.11	SKP2<br>-2.90	SLC2A1<br>2.29	SNAI1<br>1.19	SNAI2<br>1.28<br>C	SNAI3<br>-1.50	SOD1<br>-1.26	SOX10<br>1.28<br>C	STMN1<br>-4.95
G	TBX2<br>1.63	TEK<br>1.28<br>C	TEP1<br>1.26	TERF1<br>-1.48	TERF2IP<br>2.10	TINF2<br>1.69	TNKS<br>2.15	TNKS2<br>1.66	UQCRFS1<br>-1.03	VEGFC<br>-1.86	WEE1<br>-1.72	XIAP<br>2.89

40

B

MBA-MB-231

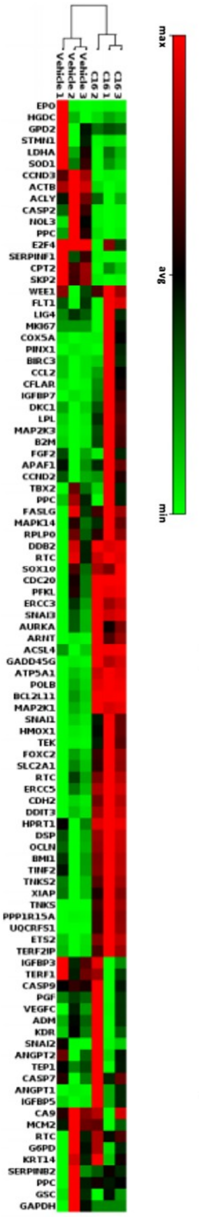

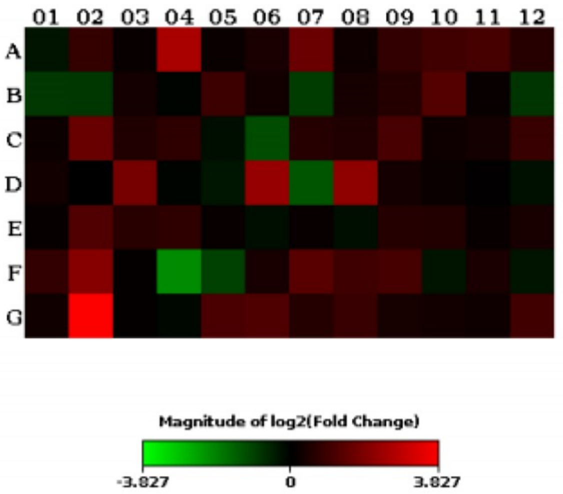

Layout	01	02	03	04	05	06	07	08	09	10	11	12
A	ACLY -1.23	ACSL4 1.70	ADM 1.08	ANGPT1 5.80 B	ANGPT2 1.11	APAF1 1.31	ARNT 3.01	ATP5A1 1.17	AURKA 1.67	BCL2L11 1.93	BIRC3 2.07	BMI1 1.49
B	CA9 -1.79 B	CASP2 -1.76	CASP7 1.24	CASP9 -1.03	CCL2 1.87 B	CCND2 1.20	CCND3 -1.85	CDC20 1.25	CDH2 1.45	CFLAR 2.40	COX5A 1.08	CPT2 -1.73
C	DDB2 1.14	DDIT3 2.94	DKC1 1.41	DSP 1.60	E2F4 -1.16	EPO -2.19 B	ERCC3 1.47	ERCC5 1.39	ETS2 2.12	FASLG 1.15 B	FGF2 1.23	FLT1 1.78 B
D	FOXC2 1.19	G6PD -1.01	GADD45 G 3.44	GPD2 -1.04	GSC -1.27 A	HMOX1 4.81	IGFBP3 -2.43 A	IGFBP5 4.44	IGFBP7 1.23	KDR 1.11 B	KRT14 1.03 B	LDHA -1.18
E	LIG4 1.07	LPL 2.31	MAP2K1 1.53	MAP2K3 1.62	MAPK14 1.08	MCM2 -1.15	MKI67 1.08	NOL3 -1.16	OCLN 1.46	PFKL 1.44	PGF 1.08	PINX1 1.29
F	POLB 1.68	PPP1R15A 4.15	SERPINB2 1.05 B	SERPINF1 -4.27 A	SKP2 -1.96	SLC2A1 1.29	SNAI1 2.51	SNAI2 1.92 B	SNAI3 2.05	SOD1 -1.23	SOX10 1.30	STMN1 -1.23
G	TBX2 1.17 B	TEK 14.19	TEP1 1.03	TERF1 -1.07	TERF2IP 2.15	TINF2 2.26	TNKS 1.45	TNKS2 1.79	UQCRFS1 1.26	VEGFC 1.22	WEE1 1.16	XIAP 1.95

C

22Rv1

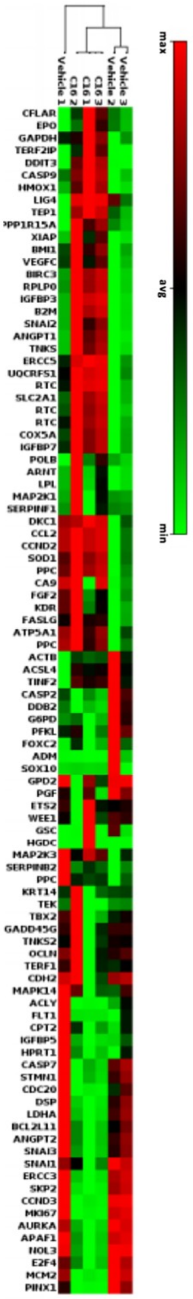

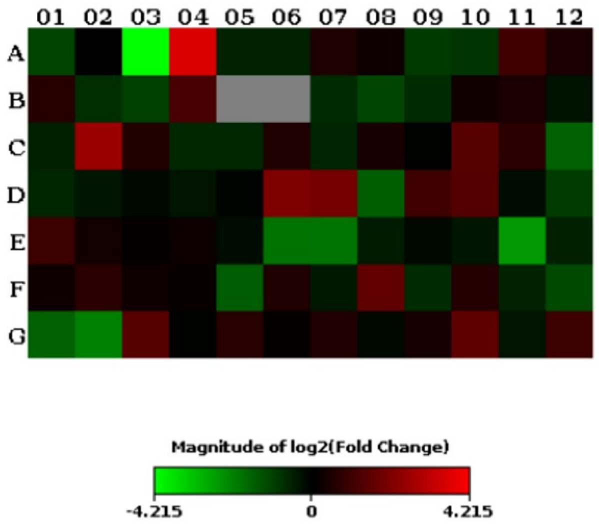

Layout	01	02	03	04	05	06	07	08	09	10	11	12
A	ACLY -2.16	ACSL4 1.00	ADM -18.57	ANGPT1 12.51	ANGPT2 -1.49	APAF1 -1.48	ARNT 1.42	ATP5A1 1.18	AURKA -1.97	BCL2L11 -1.80	BIRC3 2.08	BMI1 1.36
B	CA9 1.54 B	CASP2 -1.71	CASP7 -2.08	CASP9 2.28	CCL2 2.34 C	CCND2 2.34 C	CCND3 -1.64	CDC20 -2.23	CDH2 -1.61	CFLAR 1.20	COX5A 1.34	CPT2 -1.22
C	DDB2 -1.44	DDIT3 5.92	DKC1 1.46	DSP -1.56	E2F4 -1.58	EPO 1.41	ERCC3 -1.51	ERCC5 1.31	ETS2 -1.02	FASLG 2.68 B	FGF2 1.60	FLT1 -3.06 B
D	FOXC2 -1.54 B	G6PD -1.30	GADD45 G -1.11	GPD2 -1.25	GSC -1.04 B	HMOX1 4.38	IGFBP3 3.81	IGFBP5 -2.92	IGFBP7 2.05	KDR 2.60 B	KRT14 -1.14 A	LDHA -1.99
E	LIG4 2.00	LPL 1.22	MAP2K1 1.05	MAP2K3 1.14	MAPK14 -1.15	MCM2 -3.68	MKI67 -3.76	NOL3 -1.37	OCLN -1.09	PFKL -1.30	PGF -5.89 A	PINX1 -1.44
F	POLB 1.17	PPP1R15A 1.60	SERPINB2 1.18 B	SERPINF1 1.09	SKP2 -2.86	SLC2A1 1.42	SNAI1 -1.32 A	SNAI2 3.02	SNAI3 -1.60	SOD1 1.52	SOX10 -1.48 B	STMN1 -2.33
G	TBX2 -3.10 A	TEK -4.31 A	TEP1 2.68	TERF1 -1.03	TERF2IP 1.57	TINF2 1.05	TNKS 1.46	TNKS2 -1.10	UQCRFS1 1.30	VEGFC 2.97	WEE1 -1.27	XIAP 1.97

D

Du145

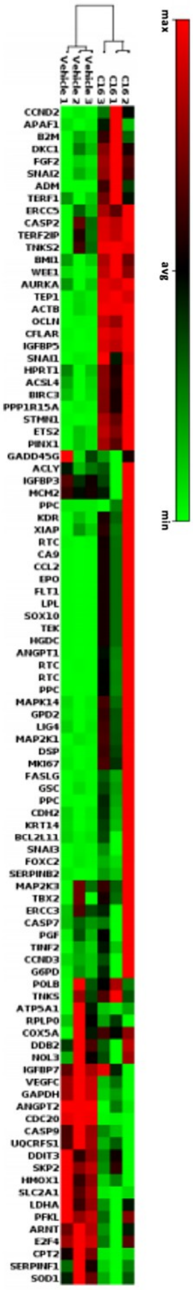

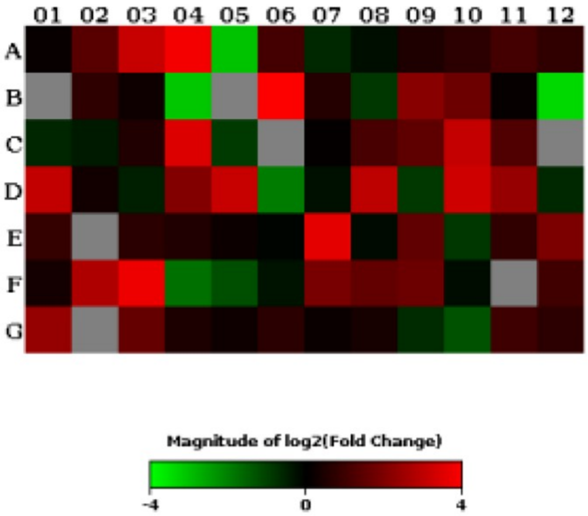

Layout	01	02	03	04	05	06	07	08	09	10	11	12
A	ACLY<br>1.10	ACSL4<br>2.60	ADM<br>8.89	ANGPT1<br>14.35<br>B	ANGPT2<br>-8.51<br>A	APAF1<br>2.09<br>A	ARNT<br>-1.54<br>A	ATP5A1<br>-1.16	AURKA<br>1.39	BCL2L11<br>1.62	BIRC3<br>2.09	BMI1<br>1.70
B	CA9<br>13.71<br>C	CASP2<br>1.63	CASP7<br>1.17<br>A	CASP9<br>-8.70<br>A	CCL2<br>13.71<br>C	CCND2<br>16.00<br>B	CCND3<br>1.47	CDC20<br>-1.81	CDH2<br>4.46<br>B	CFLAR<br>3.22	COX5A<br>1.07	CPT2<br>-10.65<br>A
C	DDB2<br>-1.50<br>A	DDIT3<br>-1.33	DKC1<br>1.41	DSP<br>11.25	E2F4<br>-1.88	EPO<br>13.71<br>C	ERCC3<br>1.04	ERCC5<br>2.21	ETS2<br>2.75	FASLG<br>8.53<br>B	FGF2<br>2.36	FLT1<br>13.71<br>C
D	FOXC2<br>8.50<br>B	G6PD<br>1.22	GADD45<br>G<br>-1.38<br>B	GPD2<br>4.16	GSC<br>8.89<br>B	HMOX1<br>-3.83<br>A	IGFBP3<br>-1.19	IGFBP5<br>7.93<br>A	IGFBP7<br>-1.84<br>A	KDR<br>9.62<br>B	KRT14<br>5.25<br>B	LDHA<br>-1.55
E	LIG4<br>1.75	LPL<br>13.71<br>C	MAP2K1<br>1.57	MAP2K3<br>1.41	MAPK14<br>1.14	MCM2<br>-1.04	MKI67<br>12.10	NOL3<br>-1.12	OCLN<br>2.84	PFKL<br>-1.81	PGF<br>1.70<br>B	PINX1<br>3.88
F	POLB<br>1.25	PPP1R15A<br>6.78	SERPINB2<br>13.50<br>B	SERPINF1<br>-3.38<br>A	SKP2<br>-2.36	SLC2A1<br>-1.22	SNAI1<br>3.71	SNAI2<br>2.91	SNAI3<br>3.21<br>A	SOD1<br>-1.15	SOX10<br>13.71<br>C	STMN1<br>2.02
G	TBX2<br>5.15<br>B	TEK<br>13.71<br>C	TEP1<br>2.99	TERF1<br>1.34	TERF2IP<br>1.17	TINF2<br>1.57	TNKS<br>1.11	TNKS2<br>1.27	UQCRFS1<br>-1.56	VEGFC<br>-2.45	WEE1<br>1.97	XIAP<br>1.62

Figure S4 – Gene expression array of cancer research molecular pathways for the (A) HTB133, (B) MDA-MB-231, (C) 22Rv1, and (D) Du145 cell lines. Abbreviations: C16, [C16Pyr][Amp].

Table S1. Half-maximal inhibitory concentration (IC50) values of Docetaxel, Doxorubicin, cyclophosphamide and paclitaxel cisplatin for different breast and prostate cell lines with indication of the duration and assay used.

Cell lines	IC_{50} **(nM)** *(duration of assay, assay used)*			
	**Docetaxel**	**Doxorubicin**	**Cyclophosphamide**	**Paclitaxel**
MCF-10A	10 (48h, MTT) [1]	100 (48h, MTT) [1]<br>2.5×10^{3} (48h, MTT) [2]	N/A	32 (72h, MTT) [3]<br>200 (24h, MTT) [4]
HTB22	250 (72h, MTT) [5]<br>1×10^{4} (72h, XTT) [6]<br>2×10^{6} (48h, MTT) [7]<br>$1\text{-}5\times10^{3}$ (48h, MTT) [8]	500 (48h, SRB) [9]<br>$1\text{-}5\times10^{3}$ (48h, MTT) [10]<br>286 (72h, MTT) [11]<br>500 (24h, MTT) [12]<br>800 (48h, MTT) [13]<br>1.1×10^{4} (72h, MTT) [14]	1×10^{7} (48h, MTT) [15]<br>1×10^{6} (96h, MTT) [16]<br>655 (72h, MTT) [14]	200 (72h, SRB) [17]<br>100-200 (48h, MTT) [10]<br>4.1×10^{3} (48h, MTT) [18]<br>3.5×10^{3} (24h, MTT) [19]<br>100 (96h, MTT) [16]
HTB133	40 (96h, MTT) [20]<br>90.56 (72h, MTT) [21]	1×10^{3} (48h, SRB) [9]<br>5×10^{4} (48h, MTT) [10]<br>293 (48h, MTT) [22]<br>2.2×10^{3} (72h, MTT) [23]<br>9.2×10^{3} (24h, MTT) [24]<br>1.8×10^{3} (72h, MTT) [25]<br>8.5×10^{3} (48h, MTT) [2]	N/A	100 (72h, SRB) [17]<br>4.8×10^{4} (48h, MTT) [18]<br>1.6×10^{3} (24h, MTT) [26]
HCC1937	$1\text{-}5\times10^{3}$ (48h, MTT) [8]<br>7.2×10^{3} (5 days, AP) [27]	3.9×10^{3} (5 days, AP) [27]<br>5×10^{4} (48h, MTT) [10]<br>1.3×10^{3} (72h, CCK-8) [28]<br>4.8×10^{3} (72h, MTT) [23]	N/A	$>2\times10^{3}$ (48h, MTT) [10]
MDA-MB-231	2.5 (24h, MTT) [29]<br>37.6 (48h, MTT) [30]<br>5×10^{3} (48h, SRB) [9]<br>3×10^{3} (5 days, AP) [27]<br>4.6×10^{4} (72h, MTT) [31]	500 (48h, SRB) [9]<br>6×10^{4} (5 days, AP) [27]<br>5×10^{4} (48h, MTT) [10]<br>138 (48h, MTT) [22]<br>160 (72h, MTT) [32]<br>280 (72h, PB) [33]<br>3×10^{3} (48h, MTT) [34]	1×10^{7} (48h, MTT) [35]<br>1.3×10^{7} (48h, MTT) [15]<br>5×10^{6} (72h, MTT) [31]	100 (48h, SRB) [9]<br>16 (72h, MTT) [3]<br>300 (24h, MTT) [19]<br>670 (48h, MTT) [34]<br>1.4×10^{3} (24h, CCK-8) [36]
RWPE	N/A	N/A	N/A	N/A
22Rv1	4 (5 days, AP) [37]<br>5 (72h, MTS) [38]<br>8 (48h, MTT) [39]	60 (5 days, AP) [37]<br>234 (48h, MTT) [40]	N/A	N/A
LNCaP	1.2 (72h, CCK-8) [41]<br>1.5 (72h, SRB) [42]<br>1.1 (48h, MTT) [43]<br>296 (72h, MTT) [44]<br>280 (72h, MTT) [45]<br>296 (72h, MTT) [46]	20 (72h, MTT) [45]<br>169 (48h, MTT) [40]<br>250 (48h, MTT) [47]<br>290 (48h, MTT) [48]<br>1.7×10^{4} (48h, MTT) [49]	N/A	1.54 (48h, trypan blue) [50]<br>22 (24h, MTT) [51]
Du145	1.7 (5 days, AP) [37]<br>5 (72h, MTS) [38]<br>17 (48h, MTT) [39]<br>25 (24h, MTT) [52]<br>2.5 (48h, MTT) [43]<br>507 (72h, MTT) [44]<br>470 (72h, MTT) [45]<br>19.3 (48h, MTT) [53]	24 (5 days, AP) [37]<br>7 (72h, MTT) [45]<br>250 (48h, MTT) [48]<br>81 (72h, MTT) [54]	N/A	5.2 (48h, trypan blue) [50]<br>5 (72h, MTT) [55]
PC-3	1.2×10^{3} (72h, MTT) [5]<br>10 (48h, MTT) [56]<br>8 (24h, MTT) [52]<br>2.1 (72h, CCK-8) [41]<br>1.1 (5 days, MTT) [57]<br>3.1 (72h, SRB) [42]<br>3.7 (48h, MTT) [43]<br>117 (72h, MTT) [44]<br>250 (72h, MTT) [45]<br>70.5 (48h, MTS) [58]<br>10 (72h, MTT) [59]<br>117 (72h, MTT) [46]	26 (72h, MTT) [45]<br>8×10^{3} (48h, MTT) [47]<br>137 (72h, MTT) [54]	N/A	13.2 (48h, MTT) [60]<br>5.2 (48h, trypan blue) [50]<br>9 (48h, MTT) [61]<br>1×10^{3} (72h, MTT) [62]<br>110 (24h, MTT) [51]

Abbreviations: AP – Acid phosphatase assay; CCK-8 – Cell Counting Kit-8, MTS – 3-(4,5-dimethylthiazol-2-yl)-5-(3-carboxymethoxyphenyl)-2-(4-sulfophenyl)-2H-tetrazolium; MTT – 3-(4,5-dimethylthiazol-2-yl)-2,5-diphenyltetrazolium-bromide assay; PB – Presto Blue Reagent; SRB – Sulfarodamine-B assay; XTT – 2,3-bis-(2-methoxy-4-nitro-5-sulfophenyl)-2H-tetrazolium-5-carboxanilide assay.

References:

1. Ozawa, P.M.M.; Alkhilaiwi, F.; Cavalli, I.J.; Malheiros, D.; de Souza Fonseca Ribeiro, E.M.; Cavalli, L.R. Extracellular vesicles from triple-negative breast cancer cells promote proliferation and drug resistance in non-tumorigenic breast cells. *Breast Cancer Research and Treatment* **2018**, *172*, 713-723, doi:10.1007/s10549-018-4925-5.
2. Wen, S.-H.; Su, S.-C.; Liou, B.-H.; Lin, C.-H.; Lee, K.-R. Sulbactam-enhanced cytotoxicity of doxorubicin in breast cancer cells. *Cancer cell international* **2018**, *18*, 128-128, doi:10.1186/s12935-018-0625-9.
3. Kumari, S.; Badana, A.K.; Mohan, G.M.; Shailender Naik, G.; Malla, R. Synergistic effects of coralyne and paclitaxel on cell migration and proliferation of breast cancer cells lines. *Biomed Pharmacother* **2017**, *91*, 436-445, doi:10.1016/j.biopha.2017.04.027.
4. Tommasi, S.; Mangia, A.; Lacalamita, R.; Bellizzi, A.; Fedele, V.; Chiriatti, A.; Thomssen, C.; Kendzierski, N.; Latorre, A.; Lorusso, V. Cytoskeleton and paclitaxel sensitivity in breast cancer: the role of β-tubulins. *International journal of cancer* **2007**, *120*, 2078-2085.
5. Gao, J.; Jiang, S.; Zhang, X.; Fu, Y.; Liu, Z. Preparation, characterization and in vitro activity of a docetaxel-albumin conjugate. *Bioorg Chem* **2019**, *83*, 154-160, doi:10.1016/j.bioorg.2018.10.032.
6. Aktas, S.H.; Akbulut, H.; Akgun, N.; Icli, F. Low dose chemotherapeutic drugs without overt cytotoxic effects decrease the secretion of VEGF by cultured human tumor cells: A tentative relationship between drug type and tumor cell type response. *Cancer Biomarkers* **2013**, *12*, 135-140, doi:10.3233/CBM-130301.
7. Ebrahimi Fard, A.; Tavakoli, M.B.; Salehi, H.; Emami, H. Synergetic effects of Docetaxel and ionizing radiation reduced cell viability on MCF-7 breast cancer cell. *Applied Cancer Research* **2017**, *37*, 29, doi:10.1186/s41241-017-0035-7.
8. Tassone, P.; Blotta, S.; Palmieri, C.; Masciari, S.; Quaresima, B.; Montagna, M.; D'Andrea, E.; Eramo, O.P.; Migale, L.; Costanzo, F. Differential sensitivity of BRCA1-mutated HCC1937 human breast cancer cells to microtubule-interfering agents. *International journal of oncology* **2005**, *26*, 1257-1263.
9. Alami, N.; Paterson, J.; Belanger, S.; Juste, S.; Grieshaber, C.K.; Leyland-Jones, B. Comparative analysis of xanafide cytotoxicity in breast cancer cell lines. *Br J Cancer* **2007**, *97*, 58-64, doi:10.1038/sj.bjc.6603829.
10. Tassone, P.; Tagliaferri, P.; Perricelli, A.; Blotta, S.; Quaresima, B.; Martelli, M.L.; Goel, A.; Barbieri, V.; Costanzo, F.; Boland, C.R., et al. BRCA1 expression modulates chemosensitivity of BRCA1-defective HCC1937 human breast cancer cells. *Br J Cancer* **2003**, *88*, 1285-1291, doi:10.1038/sj.bjc.6600859.
11. AbuHammad, S.; Zihlif, M. Gene expression alterations in doxorubicin resistant MCF7 breast cancer cell line. *Genomics* **2013**, *101*, 213-220, doi:https://doi.org/10.1016/j.ygeno.2012.11.009.
12. Barzegar, E.; Fouladdel, S.; Movahhed, T.K.; Atashpour, S.; Ghahremani, M.H.; Ostad, S.N.; Azizi, E. Effects of berberine on proliferation, cell cycle distribution and apoptosis of human breast cancer T47D and MCF7 cell lines. *Iran J Basic Med Sci* **2015**, *18*, 334-342.
13. Afzali, M.; Ghaeli, P.; Khanavi, M.; Parsa, M.; Montazeri, H.; Ghahremani, M.H.; Ostad, S.N. Non-addictive opium alkaloids selectively induce apoptosis in cancer cells compared to normal cells. *Daru* **2015**, *23*, 16-16, doi:10.1186/s40199-015-0101-1.
14. Al-Joudi, F.S.; Alias, I.Z.; Samsudin, A.-R. The effects of chemotherapeutic drugs on viabilty, apoptosis, and survivin expression in MCF-7 cells. *Acta Histochemica et Cytochemica* **2005**, *38*, 323-330.
15. Singh, N.; Nigam, M.; Ranjan, V.; Sharma, R.; Balapure, A.K.; Rath, S.K. Caspase mediated enhanced apoptotic action of cyclophosphamide-and resveratrol-treated MCF-7 cells. *Journal of pharmacological sciences* **2009**, *109*, 473-485.
16. Kern, K.M.; Schroeder, J.R. Comparison of Cantharidin Toxicity in Breast Cancer Cells to Two Common Chemotherapeutics. *International Journal of Breast Cancer* **2014**, *2014*, 423059, doi:10.1155/2014/423059.
17. Bashmail, H.A.; Alamoudi, A.A.; Noorwali, A.; Hegazy, G.A.; Ajabnoor, G.M.; Al-Abd, A.M. Thymoquinone Enhances Paclitaxel Anti-Breast Cancer Activity via Inhibiting Tumor-Associated Stem Cells Despite Apparent Mathematical Antagonism. *Molecules* **2020**, *25*, 426.
18. Lv, K.; Liu, L.; Wang, L.; Yu, J.; Liu, X.; Cheng, Y.; Dong, M.; Teng, R.; Wu, L.; Fu, P., et al. Lin28 mediates paclitaxel resistance by modulating p21, Rb and Let-7a miRNA in breast cancer cells. *PLoS One* **2012**, *7*, e40008-e40008, doi:10.1371/journal.pone.0040008.
19. Haghnavaz, N.; Asghari, F.; Elieh Ali Komi, D.; Shanehbandi, D.; Baradaran, B.; Kazemi, T. HER2 positivity may confer resistance to therapy with paclitaxel in breast cancer cell lines. *Artif Cells Nanomed Biotechnol* **2018**, *46*, 518-523, doi:10.1080/21691401.2017.1326927.
20. Esmaeili, F.; Dinarvand, R.; Ghahremani, M.H.; Amini, M.; Rouhani, H.; Sepehri, N.; Ostad, S.N.; Atyabi, F. Docetaxel-albumin conjugates: preparation, in vitro evaluation and biodistribution studies. *J Pharm Sci* **2009**, *98*, 2718-2730, doi:10.1002/jps.21599.
21. Esmaeili, F.; Dinarvand, R.; Ghahremani, M.H.; Ostad, S.N.; Esmaily, H.; Atyabi, F. Cellular cytotoxicity and in-vivo biodistribution of docetaxel poly(lactide-co-glycolide) nanoparticles. *Anticancer Drugs* **2010**, *21*, 43-52, doi:10.1097/CAD.0b013e328331f934.
22. Pichot, C.S.; Hartig, S.M.; Xia, L.; Arvanitis, C.; Monisvais, D.; Lee, F.Y.; Frost, J.A.; Corey, S.J. Dasatinib synergizes with doxorubicin to block growth, migration, and invasion of breast cancer cells. *Br J Cancer* **2009**, *101*, 38-47, doi:10.1038/sj.bjc.6605101.
23. Guin, S.; Ma, Q.; Padhye, S.; Zhou, Y.Q.; Yao, H.P.; Wang, M.H. Targeting acute hypoxic cancer cells by doxorubicin-immunoliposomes directed by monoclonal antibodies specific to RON receptor tyrosine kinase. *Cancer Chemother Pharmacol* **2011**, *67*, 1073-1083, doi:10.1007/s00280-010-1408-8.
24. Tunjung, W.A.S.; Sayekti, P.R. Apoptosis induction on human breast cancer T47D cell line by extracts of Ancorina sp. *F1000Research* **2019**, *8*.

25. Pelczynska, M.; Switalska, M.; Maciejewska, M.; Jaroszewicz, I.; Kutner, A.; Opolski, A. Antiproliferative activity of vitamin D compounds in combination with cytostatics. *Anticancer Res* **2006**, *26*, 2701-2705.
26. Rangkuti, I.Y. The Cytotoxicity of Paclitaxel Was Smaller than Doxorubicin in T47D Breast Cancer Cell. **2018**.
27. Corkery, B.; Crown, J.; Clynes, M.; O'Donovan, N. Epidermal growth factor receptor as a potential therapeutic target in triple-negative breast cancer. *Annals of Oncology* **2009**, *20*, 862-867, doi:https://doi.org/10.1093/annonc/mdn710.
28. Liu, Y.; Du, F.; Chen, W.; Yao, M.; Lv, K.; Fu, P. EIF5A2 is a novel chemoresistance gene in breast cancer. *Breast cancer* **2015**, *22*, 602-607.
29. Wang, K.; Zhu, X.; Yin, Y. Maslinic Acid Enhances Docetaxel Response in Human Docetaxel-Resistant Triple Negative Breast Carcinoma MDA-MB-231 Cells via Regulating MELK-FoxM1-ABCB1 Signaling Cascade. *Frontiers in Pharmacology* **2020**, *11*, doi:10.3389/fphar.2020.00835.
30. Dey, G.; Bharti, R.; Das, A.K.; Sen, R.; Mandal, M. Resensitization of Akt Induced Docetaxel Resistance in Breast Cancer by 'Iturin A' a Lipopeptide Molecule from Marine Bacteria Bacillus megaterium. *Scientific reports* **2017**, *7*, 17324-17324, doi:10.1038/s41598-017-17652-z.
31. Shen, M.; Duan, W.-M.; Wu, M.-Y.; Wang, W.-J.; Liu, L.; Xu, M.-D.; Zhu, J.; Li, D.-M.; Gui, Q.; Lian, L., et al. Participation of autophagy in the cytotoxicity against breast cancer cells by cisplatin. *Oncol Rep* **2015**, *34*, 359-367, doi:10.3892/or.2015.4005.
32. Alkaraki, A.; Alshaer, W.; Wehaibi, S.; Gharaibeh, L.; Abuarqoub, D.; Alqudah, D.A.; Al-Azzawi, H.; Zureigat, H.; Souleiman, M.; Awidi, A. Enhancing chemosensitivity of wild-type and drug-resistant MDA-MB-231 triple-negative breast cancer cell line to doxorubicin by silencing of STAT 3, Notch-1, and β-catenin genes. *Breast Cancer* **2020**, *27*, 989-998, doi:10.1007/s12282-020-01098-9.
33. Camirand, A.; Fadhil, I.; Luco, A.-L.; Ochietti, B.; Kremer, R.B. Enhancement of taxol, doxorubicin and zoledronate anti-proliferation action on triple-negative breast cancer cells by a PTHrP blocking monoclonal antibody. *Am J Cancer Res* **2013**, *3*, 500-508.
34. Franco, M.S.; Roque, M.C.; Oliveira, M.C. Short and Long-Term Effects of the Exposure of Breast Cancer Cell Lines to Different Ratios of Free or Co-Encapsulated Liposomal Paclitaxel and Doxorubicin. *Pharmaceutics* **2019**, *11*, 178.
35. Krishnan, A.; Hariharan, R.; Nair, S.A.; Pillai, M.R. Fluoxetine mediates G0/G1 arrest by inducing functional inhibition of cyclin dependent kinase subunit (CKS)1. *Biochem Pharmacol* **2008**, *75*, 1924-1934, doi:10.1016/j.bcp.2008.02.013.
36. Ma, J.; Fang, L.; Yang, Q.; Hibberd, S.; Du, W.W.; Wu, N.; Yang, B.B. Posttranscriptional regulation of AKT by circular RNA angiomotin-like 1 mediates chemoresistance against paclitaxel in breast cancer cells. *Aging (Albany NY)* **2019**, *11*, 11369.
37. Corcoran, C.; Rani, S.; O'Brien, K.; O'Neill, A.; Prencipe, M.; Sheikh, R.; Webb, G.; McDermott, R.; Watson, W.; Crown, J. Docetaxel-resistance in prostate cancer: evaluating associated phenotypic changes and potential for resistance transfer via exosomes. *PLoS One* **2012**, *7*, e50999.
38. Mohr, L.; Carceles-Cordon, M.; Woo, J.; Cordon-Cardo, C.; Domingo-Domenech, J.; Rodriguez-Bravo, V. Generation of Prostate Cancer Cell Models of Resistance to the Anti-mitotic Agent Docetaxel. *Journal of visualized experiments : JoVE* **2017**, 10.3791/56327, 56327, doi:10.3791/56327.
39. Orellana-Serradell, O.; Herrera, D.; Castell; #243; n, E.; Contreras, H.; #233; ctor. The transcription factor ZEB1 promotes chemoresistance in prostate cancer cell lines. *Asian J Androl* **2019**, *21*, 460-467, doi:10.4103/aja.aja_1_19.
40. Gumulec, J.; Fojtu, M.; Raudenska, M.; Sztalmachova, M.; Skotakova, A.; Vlachova, J.; Skalickova, S.; Nejdl, L.; Kopel, P.; Knopfova, L. Modulation of induced cytotoxicity of doxorubicin by using apoferritin and liposomal cages. *International journal of molecular sciences* **2014**, *15*, 22960-22977.
41. Yu, L.; Wu, X.; Chen, M.; Huang, H.; He, Y.; Wang, H.; Li, D.; Du, Z.; Zhang, K.; Goodin, S. The effects and mechanism of YK-4-279 in combination with docetaxel on prostate cancer. *International Journal of Medical Sciences* **2017**, *14*, 356.
42. Attia, R.T.; Tolba, M.F.; Trivedi, R.; Tadros, M.G.; Arafa, H.M.M.; Abdel-Naim, A.B. The chemomodulatory effects of glufosfamide on docetaxel cytotoxicity in prostate cancer cells. *PeerJ* **2016**, *4*, e2168-e2168, doi:10.7717/peerj.2168.
43. Yang, C.; Zhang, W.; Wang, J.; Chen, P.; Jin, J. Effect of docetaxel on the regulation of proliferation and apoptosis of human prostate cancer cells. *Mol Med Rep* **2019**, *19*, 3864-3870, doi:10.3892/mmr.2019.9998.
44. Mansour, M.; van Ginkel, S.; Dennis, J.C.; Mason, B.; Elhussin, I.; Abbott, K.; Pondugula, S.R.; Samuel, T.; Morrison, E. The Combination of Omega-3 Stearidonic Acid and Docetaxel Enhances Cell Death over Docetaxel Alone in Human Prostate Cancer Cells. *J Cancer* **2018**, *9*, 4536-4546, doi:10.7150/jca.26681.
45. Budman, D.R.; Calabro, A.; Kreis, W. Synergistic and antagonistic combinations of drugs in human prostate cancer cell lines in vitro. *Anticancer Drugs* **2002**, *13*, 1011-1016, doi:10.1097/00001813-200211000-00005.
46. Mansour, M.; van Ginkel, S.; Dennis, J.C.; Mason, B.; Elhussin, I.; Abbott, K.; Pondugula, S.R.; Samuel, T.; Morrison, E. The combination of omega-3 stearidonic acid and docetaxel enhances cell death over docetaxel alone in human prostate cancer cells. *J Cancer* **2018**, *9*, 4536.
47. Orzechowska, E.J.; Girstun, A.; Staron, K.; Trzcinska-Danielewicz, J. Synergy of BID with doxorubicin in the killing of cancer cells. *Oncol Rep* **2015**, *33*, 2143-2150, doi:10.3892/or.2015.3841.
48. Tehranian, N.; Sepehri, H.; Mehdipour, P.; Biramijamal, F.; Hossein-Nezhad, A.; Sarrafnejad, A.; Hajizadeh, E. Combination effect of PectaSol and Doxorubicin on viability, cell cycle arrest and apoptosis in DU-145 and LNCaP prostate cancer cell lines. *Cell Biol Int* **2012**, *36*, 601-610, doi:10.1042/cbi20110309.

49. Kim, E.; Jung, Y.; Choi, H.; Yang, J.; Suh, J.S.; Huh, Y.M.; Kim, K.; Haam, S. Prostate cancer cell death produced by the co-delivery of Bcl-xL shRNA and doxorubicin using an aptamer-conjugated polyplex. *Biomaterials* **2010**, *31*, 4592-4599, doi:10.1016/j.biomaterials.2010.02.030.
50. Li, Y.; Zeng, Y.; Mooney, S.M.; Yin, B.; Mizokami, A.; Namiki, M.; Getzenberg, R.H. Resistance to paclitaxel increases the sensitivity to other microenvironmental stresses in prostate cancer cells. *J Cell Biochem* **2011**, *112*, 2125-2137, doi:10.1002/jcb.23134.
51. Moro, L.; Arbini, A.A.; Marra, E.; Greco, M. Mitochondrial DNA depletion reduces PARP-1 levels and promotes progression of the neoplastic phenotype in prostate carcinoma. *Cell Oncol* **2008**, *30*, 307-322, doi:10.3233/clo-2008-0427.
52. Lin, J.-Z.; Wang, Z.-J.; De, W.; Zheng, M.; Xu, W.-Z.; Wu, H.-F.; Armstrong, A.; Zhu, J.-G. Targeting AXL overcomes resistance to docetaxel therapy in advanced prostate cancer. *Oncotarget* **2017**, *8*, 41064.
53. Sekino, Y.; Oue, N.; Koike, Y.; Shigematsu, Y.; Sakamoto, N.; Sentani, K.; Teishima, J.; Shiota, M.; Matsubara, A.; Yasui, W. KIFC1 inhibitor CW069 induces apoptosis and reverses resistance to docetaxel in prostate cancer. *Journal of Clinical Medicine* **2019**, *8*, 225.
54. Van Brussel, J.; Oomen, M.; Vossebeld, P.; Wiemer, E.; Sonneveld, P.; Mickisch, G. Identification of multidrug resistance-associated protein 1 and glutathione as multidrug resistance mechanisms in human prostate cancer cells: chemosensitization with leukotriene D4 antagonists and buthionine sulfoximine. *BJU international* **2004**, *93*, 1333-1338.
55. Erdogan, S.; Doganlar, O.; Doganlar, Z.B.; Turkekul, K. Naringin sensitizes human prostate cancer cells to paclitaxel therapy. *Prostate International* **2018**, *6*, 126-135, doi:https://doi.org/10.1016/j.prnil.2017.11.001.
56. O'Neill, A.J.; Prencipe, M.; Dowling, C.; Fan, Y.; Mulrane, L.; Gallagher, W.M.; O'Connor, D.; O'Connor, R.; Devery, A.; Corcoran, C. Characterisation and manipulation of docetaxel resistant prostate cancer cell lines. *Molecular cancer* **2011**, *10*, 126.
57. Lo Nigro, C.; Maffi, M.; Fischel, J.L.; Formento, P.; Milano, G.; Merlano, M. The combination of docetaxel and the somatostatin analogue lanreotide on androgen-independent docetaxel-resistant prostate cancer: experimental data. *BJU international* **2008**, *102*, 622-627.
58. Karanika, S.; Karantanos, T.; Kurosaka, S.; Wang, J.; Hirayama, T.; Yang, G.; Park, S.; Golstov, A.A.; Tanimoto, R.; Li, L., et al. GLIPR1-ΔTM synergizes with docetaxel in cell death and suppresses resistance to docetaxel in prostate cancer cells. *Molecular cancer* **2015**, *14*, 122-122, doi:10.1186/s12943-015-0395-0.
59. Hour, T.-C.; Chung, S.-D.; Kang, W.-Y.; Lin, Y.-C.; Chuang, S.-J.; Huang, A.-M.; Wu, W.-J.; Huang, S.-P.; Huang, C.-Y.; Pu, Y.-S. EGFR mediates docetaxel resistance in human castration-resistant prostate cancer through the Akt-dependent expression of ABCB1 (MDR1). *Archives of toxicology* **2015**, *89*, 591-605.
60. Zhao, Y.; Zeng, X.; Tang, H.; Ye, D.; Liu, J. Combination of metformin and paclitaxel suppresses proliferation and induces apoptosis of human prostate cancer cells via oxidative stress and targeting the mitochondria-dependent pathway. *Oncol Lett* **2019**, *17*, 4277-4284, doi:10.3892/ol.2019.10119.
61. Byun, W.S.; Jin, M.; Yu, J.; Kim, W.K.; Song, J.; Chung, H.-J.; Jeong, L.S.; Lee, S.K. A novel selenonucleoside suppresses tumor growth by targeting Skp2 degradation in paclitaxel-resistant prostate cancer. *Biochemical Pharmacology* **2018**, *158*, 84-94, doi:https://doi.org/10.1016/j.bcp.2018.10.002.
62. Ping, S.Y.; Hour, T.C.; Lin, S.R.; Yu, D.S. Taxol synergizes with antioxidants in inhibiting hormal refractory prostate cancer cell growth. *Urologic oncology* **2010**, *28*, 170-179, doi:10.1016/j.urolonc.2008.07.003.

Synthesis and spectral of 1-Hexadecylpyridin-1-ium (2S,5R,6R)-6-((R)-2-amino-2-phenylacetamido)-3,3-dimethyl-7-oxo-4-thia-1-azabicyclo[3.2.0]heptane-2-carboxylate [C16Pyr][Amp]

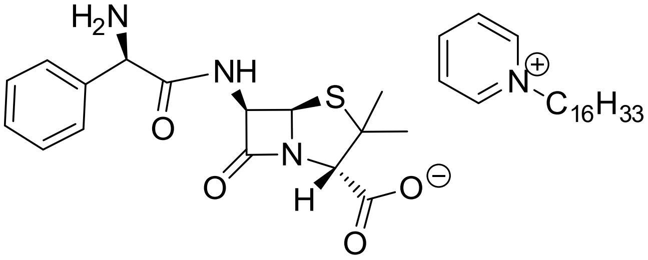

Struture of [C16Pyr][Amp]

Cetylpyridinium chloride (0.694 g; 2.04 mmol), was dissolved in methanol and passed through an ion-exchange column Amberlite IRA-400-OH[26,30] (5 eq., flux rate 0.133 mLmL^{-1}min^{-1} = 8 BVh^{-1}). Then, cetylpyridinium hydroxide solution was slowly added to ampicillin (0.714 g; 2.12 mmol) dissolved in 1.0 M ammonium solution (50 mgmL^{-1}). The mixture was stirred at room temperature for 1 h. After solvent evaporation the residue was dissolved in 20 mL of (methanol/acetonitrile 1:9)[26,30] and left refrigerated overnight (4 ºC)[30] to induce crystallization of ampicillin excess. Then, ampicillin crystals were filtered from the solution which was evaporated and dried *in vacuum* for 24h. The desired product was obtained as a yellow solid (1.018 g; 76.4 %). m.p. 86ºC; $[\alpha]_D^{27}$= 51.7 ± 0.9 (c = 2 mgmL^{-1} in methanol); ^{1}H-NMR (400.13 MHz, CD_3OD) δ = 8.98 (2H, d, *J* = 5.5Hz), 8.58 (1H, t, *J* = 7.8Hz), 8.10 (2H, t, *J* = 6.70Hz) 7.47 (2H, d, *J* = 7.3Hz), 7.35 (2H, t, *J* = 7.4Hz), 7.28 (1H, d, *J* = 7.3Hz), 5.0 (1H, d, *J* = 6.0 Hz), 4.62 (3H,m), 4.33 (1H, d, *J* = 6.0), 3.43 (1H, s), 2.01 (2H, m), 1.65-1.11 (32H), 0.90 (3H, t, *J* = 6.7 Hz) ppm; ^{13}C-NMR (100.62MHz, CD_3OD) δ = 175.52, 174.80 , 170.23, 146.87, 145.93, 129.88, 129.78, 129.76, 129.72, 129.56, 129.02, 128.59, 77.15, 66.65, 63.15, 62.40, 61.41, 60,18, 59.55, 33.09, 32.52, 30.80, 30.78, 30.74, 30.65, 30.53, 30.49, 30.15, 27.78, 27.45, 27.73, 23.76, 14.48 ppm; IR (KBr): ν = 3419, 3061, 2923, 2852, 1688, 1593, 1483, 1456, 1385, 1176, 1130, 1029, 964, 778, 686 cm^{-1}; (EI$^+$) *m/z* calcd for $C_{21}H_{38}N^+$: 304.2999, found 304.2999; (EI$^-$) *m/z* calcd for $C_{16}H_{18}N_3O_4S^-$: 348.1024, found 348.1013.

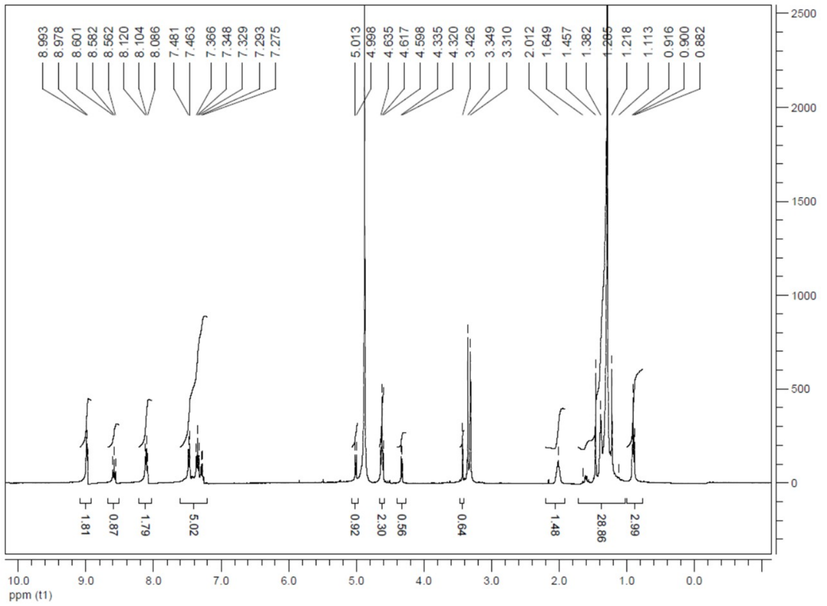

[C_{16}Pyr][Amp] ^{1}H-NMR spectrum in CD_3OD.

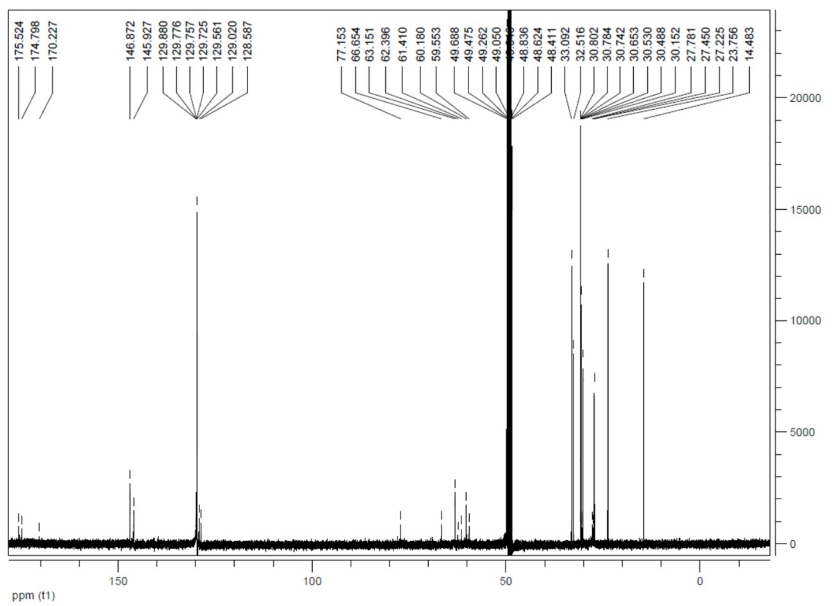

[C_{16}Pyr][Amp] ^{13}C-NMR spectrum in CD_3OD.

Synthesis and spectral data of 3-(2-Hydroxyethyl)-1-methyl-1*H*-imidazol-3-ium (2S,5R,6R)-6-((R)-2-amino-2-phenylacetamido)-3,3-dimethyl-7-oxo-4-thia-1-azabicyclo[3.2.0]heptane-2-carboxylate [C_2OHMIM][Amp]

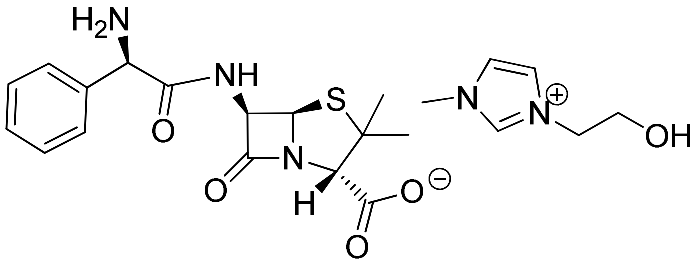

Struture of [C_2OHMIM][Amp].

3-(2-Hydroxyethyl)-1-methyl-1*H*-imidazol-3-ium chloride (0.625 g; 3.86 mmol) was dissolved in methanol and passed through an ion-exchange column Amberlite IRA-400-OH[26,30] (5 eq., flux rate 0.133 $mLmL^{-1}min^{-1}$ = 8 BVh^{-1}). Then the hydroxide solution formed was slowly added to Ampicillin (1.624 g; 4.65 mmol; 1.2 eq) dissolved in 1.0 M ammonium solution (50 $mgmL^{-1}$). The mixture was stirred at room temperature for 1 h. After solvent evaporation, the residue was dissolved in 20 mL of (methanol/acetonitrile 1:9)[26,30] and left refrigerated overnight (4 ^{o}C)[30] to induce crystallization of excess of ampicillin. Then, ampicillin crystals were filtered from the solution which was evaporated and dried *in vacuum* for 24 h. The desired product was obtained as a yellow solid (1.593 g; 86.8 %). m.p. 115-117 ^{o}C; $[\alpha]_D^{26}$= 86.3 ± 4.5 (c = 2 $mgmL^{-1}$ in methanol); ^{1}H-NMR (400.13 MHz, CD_3OD) δ = 7.61 (1H, d, *J*= 1.8Hz, k), 7.55 (1H, d, *J* = 1.8Hz, j), 7.47 (2H, d, *J* = 7.2Hz, m), 7.35 (2H, t, *J* = 7.3Hz,l), 7.29 (1H, d, *J* = 7.2Hz, n), 5.00 (1H, d, *J* =6.0 Hz, c), 4.63 (1H, s, b), 4.33 (1H, d, *J* =6.0 Hz, d), 4.28 (2H, t, *J* =3.8 Hz, g), 3.92 (3H, s, f), 3.87 (2H, t, *J*=3.8 Hz), 3.42 (1H, s), 1.45 (3H, s), 1.22 (3H, s) ppm; ^{13}C-NMR (100.62 MHz, CD_3OD) δ = 175.60, 175.17, 174.85, 141.22, 129.85, 129.14, 128.51, 124.74, 124.03, 77.11, 66.67, 61.06, 60.19, 60.12, 59.53, 53.29, 36.46, 27.78, 27.48 ppm; IR (KBr): ν = 3394, 2969, 2888, 2836, 1674, 1545, 1456, 1394, 1299, 1253, 1167, 1131, 1073, 1027, 1071, 871, 784, 752, 702, 652, 622 cm^{-1}; (EI^+) *m/z* calcd for $C_6H_{11}N_2O^+$: 127.0866, found 127.0866; (EI^-) *m/z* calcd for $C_{16}H_{18}N_3O_4S^-$: 348.1024, found 348.1013.

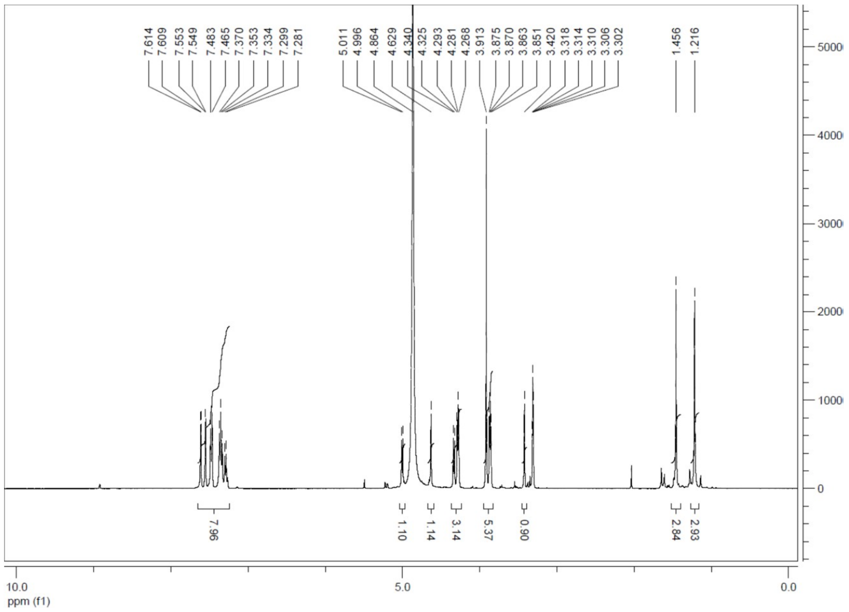

[C2OHMIM][Amp] ^{1}H-NMR spectrum in CD_3OD.

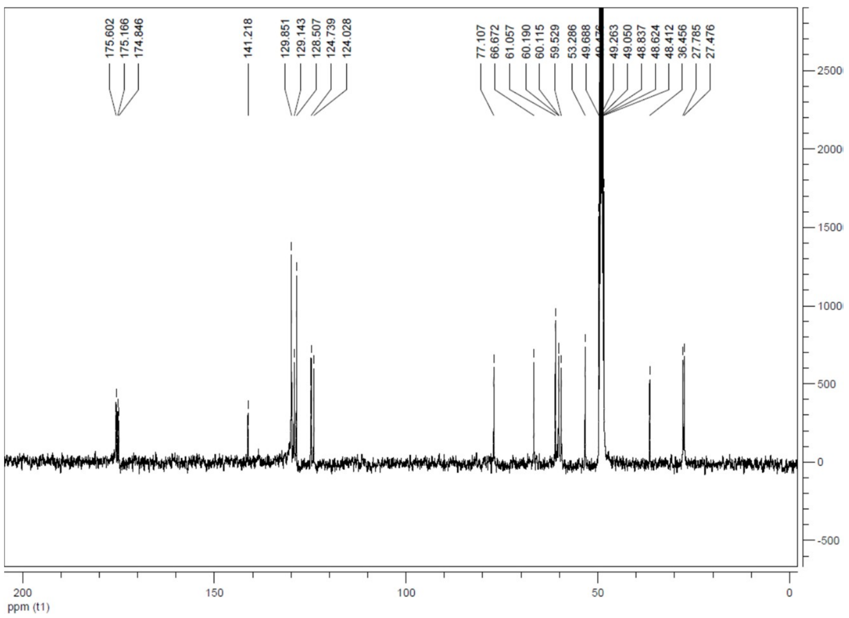

[C2OHMIM][Amp] ^{13}C-NMR spectrum in CD_3OD.
